# Memory acquisition and retrieval impact different epigenetic processes that regulate gene expression

**DOI:** 10.1186/1471-2164-16-S5-S5

**Published:** 2015-05-26

**Authors:** Lucia L Peixoto, Mathieu E Wimmer, Shane G Poplawski, Jennifer C Tudor, Charles A Kenworthy, Shichong Liu, Keiko Mizuno, Benjamin A Garcia, Nancy R Zhang, K Peter Giese, Ted Abel

**Affiliations:** 1 Department of Biology, University of Pennsylvania, Smilow Center for Translational Research, Room 10-170, Building 421, 3400 Civic Center Boulevard, Philadelphia, PA 19104-6168, USA; 2 Department of Psychiatry, University of Pennsylvania,125 S, 31st street, suite 1102A, Philadelphia, PA 19104, USA; 3 Department of Anatomy and Structural Biology, Albert Einstein College of Medicine, 1300 Morris Park Avenue NY, USA; 4 Epigenetics Program, Department of Biochemistry and Biophysics, University of Pennsylvania, 9-124 Smilow Center for Translational Research, 3400 Civic Center Blvd, Philadelphia, PA 19104-6059, USA; 5 Centre for the Cellular Basis of Behaviour, King's College London, 125 Coldharbour Lane, London, SE5 9NU, UK; 6 Department of Statistics, Wharton School, University of Pennsylvania, 3730 Walnut Street, Suite 467, Philadelphia, PA 19104, USA; 7 Department of Biology, University of Pennsylvania, Smilow Center for Translational Research, Room 10-133, Building 421, 3400 Civic Center Boulevard, Philadelphia, PA 19104-6168, USA

**Keywords:** Learning and memory, gene expression, normalization of unwanted variance, histone variants, microRNAs

## Abstract

**Background:**

A fundamental question in neuroscience is how memories are stored and retrieved in the brain. Long-term memory formation requires transcription, translation and epigenetic processes that control gene expression. Thus, characterizing genome-wide the transcriptional changes that occur after memory acquisition and retrieval is of broad interest and importance. Genome-wide technologies are commonly used to interrogate transcriptional changes in discovery-based approaches. Their ability to increase scientific insight beyond traditional candidate gene approaches, however, is usually hindered by batch effects and other sources of unwanted variation, which are particularly hard to control in the study of brain and behavior.

**Results:**

We examined genome-wide gene expression after contextual conditioning in the mouse hippocampus, a brain region essential for learning and memory, at all the time-points in which inhibiting transcription has been shown to impair memory formation. We show that most of the variance in gene expression is not due to conditioning and that by removing unwanted variance through additional normalization we are able provide novel biological insights. In particular, we show that genes downregulated by memory acquisition and retrieval impact different functions: chromatin assembly and RNA processing, respectively. Levels of histone 2A variant H2AB are reduced only following acquisition, a finding we confirmed using quantitative proteomics. On the other hand, splicing factor *Rbfox1 *and NMDA receptor-dependent microRNA *miR-219 *are only downregulated after retrieval, accompanied by an increase in protein levels of *miR-219 *target CAMKIIγ.

**Conclusions:**

We provide a thorough characterization of coding and non-coding gene expression during long-term memory formation. We demonstrate that unwanted variance dominates the signal in transcriptional studies of learning and memory and introduce the removal of unwanted variance through normalization as a necessary step for the analysis of genome-wide transcriptional studies in the context of brain and behavior. We show for the first time that histone variants are downregulated after memory acquisition, and splicing factors and microRNAs after memory retrieval. Our results provide mechanistic insights into the molecular basis of cognition by highlighting the differential involvement of epigenetic mechanisms, such as histone variants and post-transcriptional RNA regulation, after acquisition and retrieval of memory.

## Background

Genome-wide differential gene expression analysis is widely used in discovery-based studies in biology and medicine. The question of how variability impacts reproducibility of genome-wide results has been subject to extensive research [1]. It is known that unwanted variation is often a confounding factor. Unwanted variation refers to other factors that influence the observed gene expression levels besides the one of interest. A typical example is a batch effect, which can occur when some samples are processed differently than others. Batch effects are not the only source of unwanted variance. Unwanted variance in microarrays arising from technical aspects of the methodology is removed using normalization methods such as RMA [2]. The amount of unwanted biological variance depends on the question of interest and is influenced by factors such as heterogeneity in cell-types, variability in responsiveness to stimulus between biological replicates and the simultaneous presence of other stimuli other than the one of interest, such as time of day or other environmental variables. All of these factors are present when studying gene expression in the brain *in vivo *and are often hard to control. Thus, in the context of brain and behavior a major challenge is to normalize unwanted variation to minimize false discoveries, increase resolution and maximize the potential of discovery-based approaches to contribute biological insight.

Several aspects of brain function are linked to transcriptional changes. Long-term memory formation, for example, is known to require transcription, protein synthesis and epigenetic processes that regulate gene expression [3-8]. How memories are stored and retrieved in the brain is a fundamental question in neuroscience. Thus, characterizing genome-wide the transcriptional changes that occur after memory acquisition and retrieval is of broad interest and importance. Research has shown that there are "sensitive periods" after memory acquisition during which inhibiting mRNA or protein synthesis impairs memory formation. Using contextual fear conditioning as a task, these windows occur immediately or 4 hours after acquisition for memory tested 24 hours later [9,10], or 12 hours after training for memory tested a week later [11]. Processes that follow retrieval of the memory trace (extinction or reconsolidation) also require transcription and protein synthesis [12-15]. Several studies have used genome-wide approaches such as microarrays to describe changes in coding and non-coding gene expression after memory acquisition or synaptic activity [16-20]. These studies have led to the identification of some genes relevant for memory formation, such as *c-rel *or *miR-182 *[18,21]. It remains unclear to what degree the variety of other stimuli experienced by the brain *in vivo *hinders reproducibility and limits the applicability of genome-wide technologies to the study of the brain and behavior.

Here, we examined genome-wide gene expression after contextual conditioning in the mouse hippocampus, a brain region essential for memory formation, during all the established sensitive periods for transcriptional inhibition. We show that most of the variance in gene expression is not due to conditioning and that by removing unwanted variance through additional normalization we are able provide novel biological insights. We show for the first time that histone variants are downregulated after memory acquisition, and splicing factors and microRNAs after retrieval. Our results provide mechanistic insights into the molecular basis of cognition by highlighting the differential involvement of epigenetic mechanisms, such as histone variants and post-transcriptional RNA regulation, after acquisition and retrieval of memory.

## Results and discussion

We examined genome-wide changes of gene expression in adult, male C57BL6/J mice following a contextual fear conditioning paradigm (FC), a form of learning in which an aversive stimulus (e.g a shock, US) is associated with a neutral context (CS). Re-exposure to the context triggers retrieval of the memory for the context-shock association (CS-US), which is quantified as freezing in mice. FC is highly reproducible among individuals, requiring a single exposure to the CS-US pairing to learn. In addition the timeline of sensitivity for transcriptional inhibition is established, making it an ideal learning task for our genomic study. FC is known to require the hippocampus, a brain region essential for long-term memory formation. Hippocampal tissue was collected at the established sensitive periods for transcriptional inhibition during memory consolidation: 30 minutes (FC30'), 4 hours (FC4), 12 hours (FC12) or 24 hours after FC (FC24), as well as 30 minutes after memory retrieval (RT30'). Animals in the retrieval group showed typical learning of the task, with average freezing of 55% (+/- 10%) after re-exposure to the context. Animals that were handled but not trained were dissected at the same time of day to control for circadian variation in gene expression (CC30', CC4 and CC12). RNA from nine animals per group representing nine independent behavioral experiments conducted at the same time of day (72 samples) was hybridized simultaneously to an Affymetrix gene Titan Mouse 1.1 gene-EST microarray. Pairing of the CS and US was necessary to evaluate proper retrieval of the memory trace. We have previously shown that genome-wide gene expression changes in the hippocampus 30 minutes after exposure to the CS alone are not different from those after CS+US pairing [17,22]. These results suggest that in this brain region the US alone does not produce significant differences in gene expression and that differences in gene expression observed are likely due to the encoding of the spatial memory for the context alone. Therefore, we did not include animals exposed to only the CS or US in the design.

### Individual variability and circadian time are the biggest drivers of variance in gene expression in the hippocampus *in vivo*

To explore the main sources of variance in the data, we first performed a principal component analysis (PCA) [23] (Additional file 1). None of the first three principal components (PC), which account for over 65% of the variance, capture the response to the treatment. Understanding the dominant sources of variance is critical to accurately assess the effects of learning in gene expression. The first and second principal components (PC1 and PC2) represent unusual variability in gene expression in individual mice (Additional file 1A and 1B). Interestingly, the contribution of PC1 and PC2 to variance in gene expression is correlated for a subset of genes (Additional file 1C) and may represent the same biological process. Functional annotation analysis of the genes with correlated scores between PC1 and PC2 shows that they represent response to olfactory stimuli, specifically pheromones (Additional file 2), suggesting that individual responses to, or environmental variations in olfactory stimuli are the strongest drivers of differences in gene expression.

There is substantial evidence that memory consolidation is affected by circadian time [24-26]. However, the circadian influence on genome-wide gene expression in the mouse hippocampus is poorly understood. Published genome-wide studies of gene expression in response to activity in the brain that include several time points often do not include controls for time of day [16,18], and thus it is hard to distinguish the effect of neuronal activity from the circadian effect in such studies. In our analysis, the third principal component (PC3) reveals that circadian time has a strong influence on hippocampal gene expression. Additional file 1D shows that the effect of circadian is similar to the effect of learning at the second (4 h) and third (12 h) sensitive periods, but not immediately after (30 minutes) memory acquisition or retrieval. To characterize gene expression changes in the mouse hippocampus due to circadian time, we compared genome-wide gene expression among our three control time-points (CC30', CC4 and CC12). The greatest number of differences in gene expression was detected between CC30 and CC12. These time points correspond to Zeitgeber times 3 and 15 (ZT3 and ZT15), one time-point during the light phase and one during the dark phase. 1067 probe sets, corresponding to 1019 known genes, were differentially expressed at a false discovery rate (fdr) <0.1. (Additional file 3). To determine whether our dataset corresponds to known circadian regulated genes, we compared it to those genes known to oscillate in the mouse liver according to Hughes and colleagues [27]. Of the 1019 genes regulated by time of day in the hippocampus, 198 oscillate with 24 hour periodicity in the mouse liver (Additional file 4A). This overlap is significantly higher than expected by chance (Fisher's exact p-value of 0.004) based on an expected 15% overlap between any 2 mouse tissues as reported in the mouse gene atlas [28]). Our dataset is the first genome-wide dataset describing the effects of circadian time on gene expression in the hippocampus. Genes differentially expressed between ZT3 and ZT15 in the hippocampus include *Per1*, *Per2 *and *Per3*, which are known circadian oscillators [27]. Interestingly, genes usually thought to be associated with memory formation, such as *Arc*, *Bdnf *[29,30], *CBP *[31-35] and *p300 *[36,37] also show circadian changes in expression (Additional file 4B).

### Memory acquisition and retrieval induce similar, but distinct, genome-wide changes in gene expression 30 minutes after exposure

**Figure 1:**
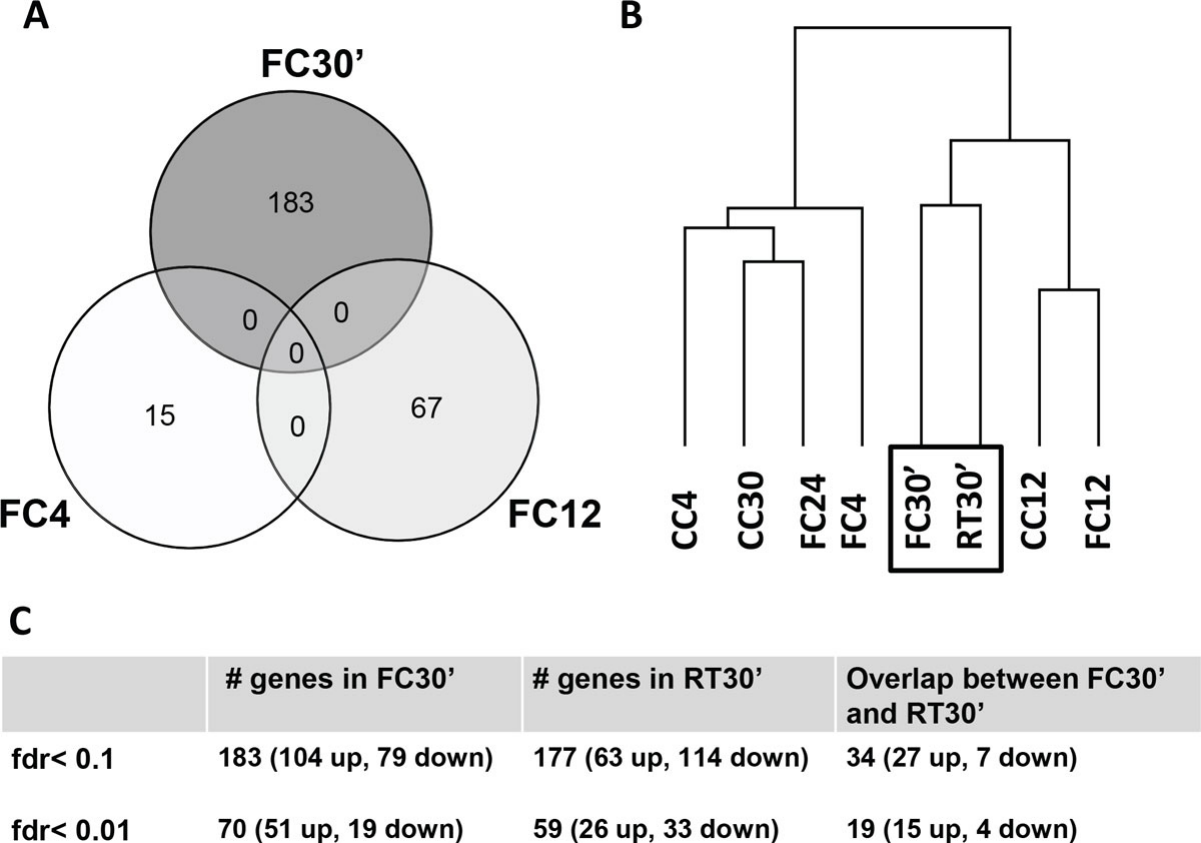
**Genome-wide changes in gene expression after memory acquisition and retrieval**. **A**. Number of probe sets differentially expressed at each sensitive period during memory acquisition (FC30', FC4 and FC12) relative to their circadian time controls (CC30', CC4, CC12). **B**. Genome-wide hierarchical clustering of gene expression patterns at all time-points. Changes 30 minutes after acquisition (FC30') and 30 minutes after retrieval (RT30') are similar genome-wide (black box). **C**. Number of differentially regulated genes at FC30 and RT30 at two different false discovery rates (fdr <0.01 and < 0.1) and their overlap. The overlap is concentrated in upregulated genes.

To accurately assess the effect of contextual conditioning in gene expression in the hippocampus, we first removed unwanted variation detected by PCA by normalizing the expression matrix using k = 1 PCs, as described in experimental procedures. Subsequently, differential expression analysis was only carried out in comparison to time of day matched controls, to ensure that circadian time was not a confounding variable in the analysis. In addition to increase power, we implemented local false discovery rates (fdr) based on empirical null hypothesis estimation to account for multiple testing [38]. Local fdr estimation provides advantages above traditionally used Benjamini and Hochberg fdr correction [39] in cases in which the null distribution can be easily estimated from the data, or in other words in datasets in which the majority of the genes are not differentially expressed due to the treatment such as ours. After normalization, we observe the greatest number of gene expression changes at the first sensitive period during memory consolidation (FC30'): 183 probe sets, representing 126 known genes (fdr <0.1). The number of genes whose expression changed at the two other sensitive periods (FC4 and FC12) was small and non-overlapping (Figure 1A). No changes in gene expression were detected 24 hours after training. We cannot distinguish which transcriptional changes correspond to the memory for the context alone and which ones to the memory for the context with the shock. We have previously shown, however, that gene expression changes detected in the hippocampus 30 minutes after FC using microarrays are not significantly different from those induced by context alone [17]. Thus it is possible that the signal-to-noise resolution in genome-wide studies is not enough to differentiate the transcriptional responses between those two memory traces. Hierarchical clustering revealed that genome-wide changes immediately following acquisition (FC30') were similar to changes observed after retrieval of memory (RT30') (Figure 1B). Additional file 5 summarizes the results of the effects of contextual conditioning in the mouse hippocampus. The number of genes regulated at FC30' and RT30' is shown in Figure 1C at two different false discovery rates, fdr<0.1 and <0.01. Fold changes observed through microarrays are small. Our use of whole hippocampal homogenates will dilute the signal when only a small proportion of the cells in the sample (such as neurons) are responsible for the changes, and thus statistically robust differences in expression that appear small in magnitude are expected.

**Figure 2:**
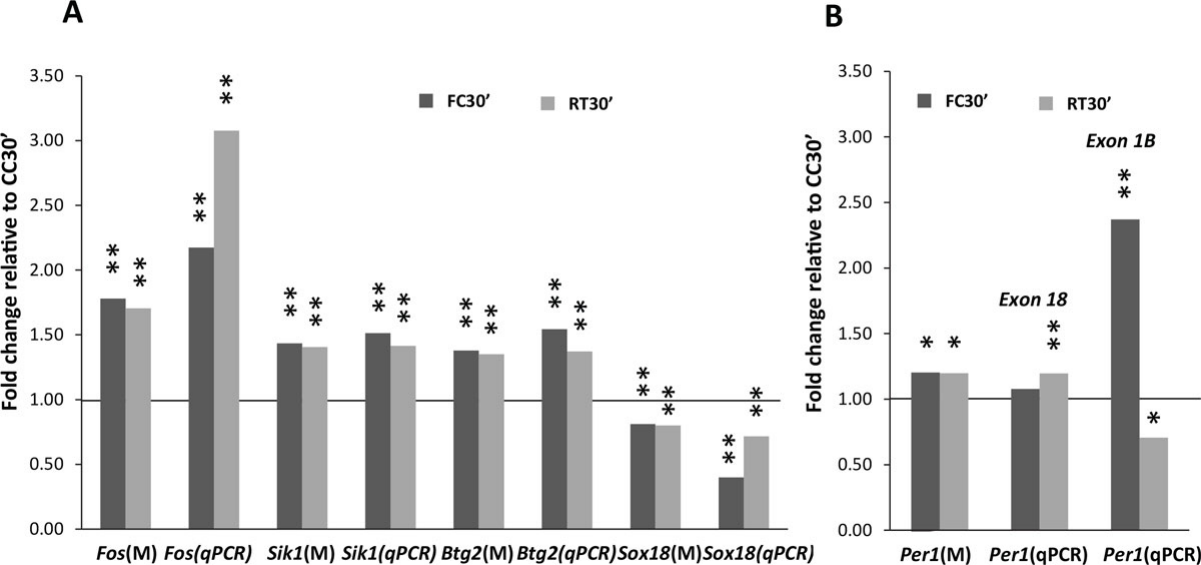
**Memory acquisition and retrieval upregulate the expression of similar genes**. **A**. *Btg2*, *Sox18 *and *Sik1 *are regulated at both FC30' (dark grey) and RT30' (light gray). **B**. Selective upregulation of *Per1 *isoforms at FC30' and RT30'. Microarray and qPCR samples are from independent cohorts of animals (*n *= 9, *n *= 8). qPCR values represent average fold change relative to controls (CC30') after normalization against *Gapdh *levels. Microarray experiment results are labeled (M). *Fos *is included as positive control. Statistical significance is shown as either fer (microarrays) or p-values (qPCR), <0.01(**) or <0.1 (*).

Several genes not previously reported to be regulated by memory acquisition and retrieval in the hippocampus were identified and validated by qPCR in a new cohort of animals (*n *= 8). We show that log fold-changes as small as 0.1 in microarrays are reproducible by qPCR in an independent set of experiments. Changes in gene expression that are similar between FC30' and RT30' include the induction of *Btg2 *and *Sik1*, as well as the downregulation of *Sox18 *(Figure 2A). The potential role of these identified gene candidates in learning and memory can be largely substantiated by available literature. *Btg2 *is a pan-neural gene whose deletion or overexpression has been shown to alter contextual memory [40]. It has been previously shown to be induced in the amygdala after contextual conditioning [17], but not in the hippocampus. Sox18 is known to interact with MEF2, an important regulator of neuronal differentiation [41] and hippocampal learning [42]. *Sik1*, another candidate gene identified in our study, also affects MEF2 activity as well as being a repressor of CREB transcription through CRTC [43-46]. CREB-dependent and MEF2-dependent transcription are both known to be important for long-term memory formation. Memory acquisition and retrieval also induce *Per1 *expression; however they differentially affect expression of the two known isoforms (NM_011065 and NM_001159367). Indeed Per1 knockout mice have been shown to have hippocampal-dependent memory deficits [47]. Measuring expression of exon18 which is present in both isoforms by qPCR shows an increase only at RT30'. However, measuring only expression of isoform 1 (exon 1B) shows a sharp increase at FC30' and a reduction at RT30' (Figure 2B), suggesting that both processes differentially affect *Per1 *splicing. We also confirmed induction of a majority of genes previously shown to be upregulated after memory acquisition and reproduced in several studies. These include *Arc*, *Fos*, *Fosb*, *Dusp1*, *Egr1 *(*Zif268*), *Egr2*, *Nr4a1*, *JunB*, *Sgk1, Npas4, Ddit4 *and *Nfkbia*. [17-19,48], all of which are also upregulated after retrieval (Additional file 5). We do not observe induction of *Bdnf *at FC30' in our microarray analysis or by qPCR analysis of levels of individual *Bdnf *isoforms (data not shown), contrary to what has been previously reported [49]. *Bdnf *induction does not peak until 2 hours after fear conditioning [49], thus levels of *Bdnf *may not have increased sufficiently for detection in our samples.

**Figure 3:**
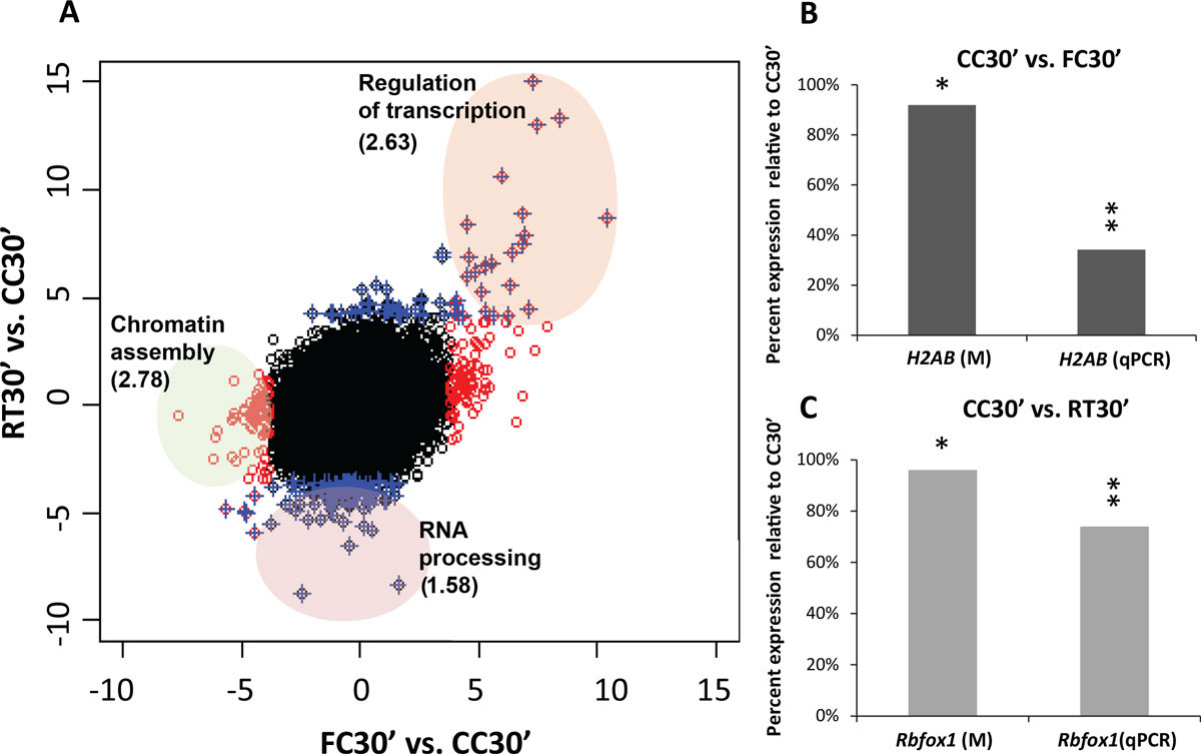
**Memory acquisition and retrieval down-regulate different processes**. **A**. Comparison of differential expression between memory acquisition (x-axis) and retrieval (y-axis). Values represent t-statistics of each condition (FC30', RT30') vs. circadian time control (CC30') for each gene. Genes not differentially expressed are represented as black circles, differentially expressed (DE) genes at FDR <0.1 are represented as red circles for FC30 and blue crosses for RT30'. Results of functional annotation clustering analysis (**Figures S4 and S5**) for each class of DE genes are shown in colored ovals and labeled according to enriched function. Genes upregulated at FC30' and RT30' corresponding to the function "Regulation of transcription" are highlighted in orange. Genes downregulated at FC30 corresponding to the function "Chromatin assembly" are shown in green. Genes downregulated at RT30' corresponding to the function "RNA processing" are shown in pink. Enrichment scores (EASE) for functional clusters are shown in parenthesis, all functions are significantly enriched compared to the expected frequency for genes in the microarray (EASE >1.3 ~ geometric mean of p-value of all functions in the cluster <0.05) **B**. Downregulation of gene expression of *H2AB *at the first sensitive period during consolidation (FC30', dark grey) **C**. Downregulation of gene expression of *Rbfox1 *after retrieval of memory (RT30', light gray). Microarray and qPCR samples belong to independent cohorts of animals (*n *= 9, *n *= 8). Values represent average fold change relative to controls (CC30') after normalization against *Gapdh *levels. Microarray experiment results are labeled: (M). Statistical significance is shown as either fdr (microarrays) or p-values (qPCR) <0.05 (**) or <0.1 (*).

To investigate which functions or pathways are affected by memory acquisition and retrieval, we carried out functional annotation and functional interaction analyses of protein coding genes. Figure 3A depicts the relationship between genes regulated in FC30' and RT30' and the corresponding functional categories. The scatterplot shows t-statistics of differential expression relative to CC30' for both groups plotted relative to each other. Genes that are statistically significant in the FC30' vs. CC30' comparison at fdr <0.1 are shown in red, and those significant in the RT30' vs. CC30' comparison in blue. The upper-right quadrant highlights genes that are up-regulated in both conditions. Interestingly, the overlap between FC30' and RT30' concentrated in upregulated genes shows enrichment for a single functional class: transcriptional regulation (Figure 3A), and includes genes such as *Btg2*, *Fos*, *Egr1*, *Egr2*, *Nr4a1*, *JunB*, and *NfKbia*. Further detail on the results of the functional annotation analysis can be found in Additional files 6 and 7. To identify the regulatory networks involved the regulation of this class of genes; we performed functional interaction network analysis of up-regulated genes at both time-points (Additional file 8A). The results suggest that three main transcriptional networks are being activated by both memory acquisition and retrieval, all highly interconnected: a MAPK/CREB network, an Nf-κB network, as well as a network represented by *Per1 *(Additional file 8B). These results agree with a previously established role of the MAPK/CREB and Nf-κB transcriptional pathways in learning and memory formation [50,51], and thus provides further evidence supporting the robustness of our approach.

### Memory acquisition and retrieval downregulate different epigenetic processes that modify gene expression

An interesting observation in Figure 3A is that the lower-left quadrant is almost empty, showing little overlap between downregulated genes. Accordingly, genes downregulated after acquisition and retrieval show no overlap in function. Chromatin assembly is downregulated after acquisition (Additional file 7A), exemplified by histone 2A isoforms *Hist1h2af*, *Hist2h2ab*, *Hist1h2ao *and *Hist2h2aa1*. RNA processing is downregulated after retrieval (Additional data file 7B), exemplified by splicing factors *Prpf38b *and *Rbfox1*, and spliceosome kinase *Srpk2*. Downregulation of genes involved in chromatin assembly observed in our microarray after acquisition is driven by downregulation of Histone 2A isoforms. Based on closer inspection of the probe-level data, *Hist2h2ab *emerged as the H2A gene most likely regulated by during memory consolidation. Greater than 2-fold downregulation of expression of *Hist2h2ab*, herein referred to as *H2AB*, was confirmed by qPCR in an independent cohort of animals (Figure 3B).

**Figure 4:**
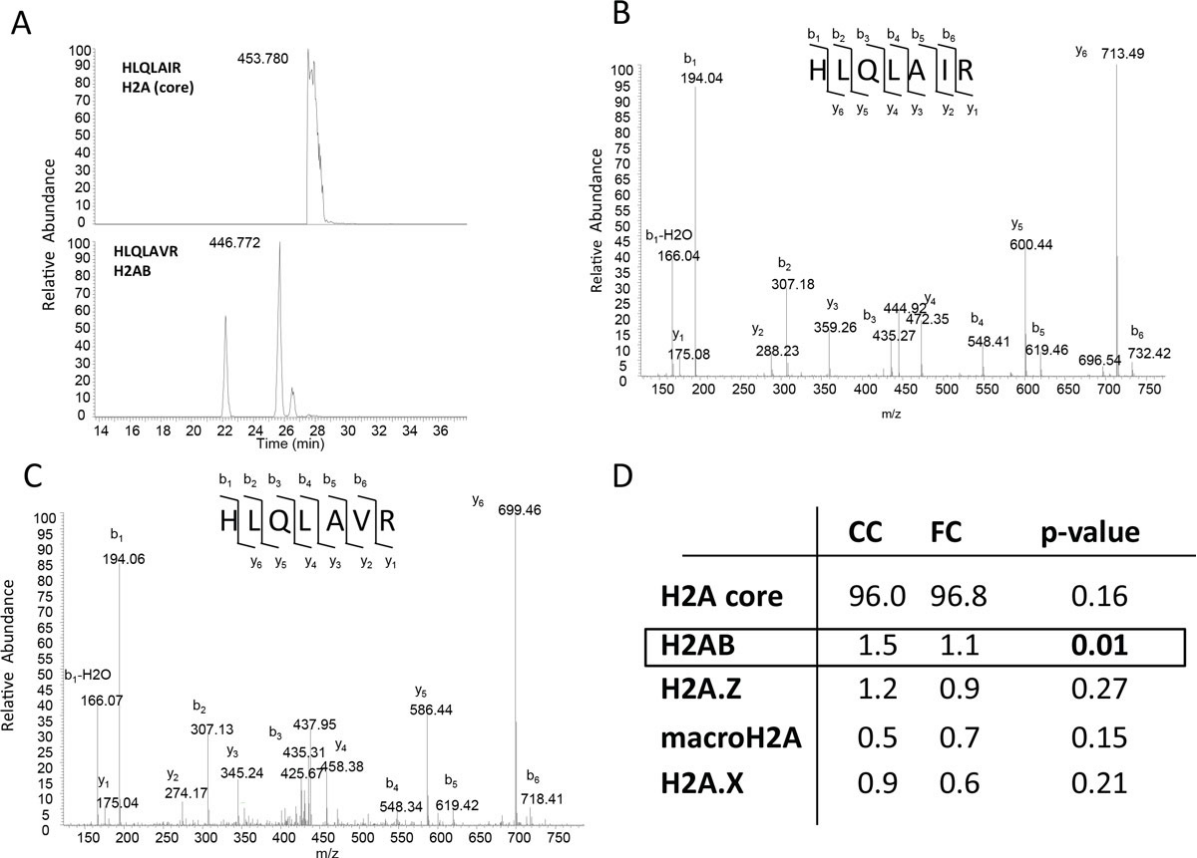
**Quantitative proteomic analysis of histone variants following contextual conditioning**. **A**. Single ion chromatograms of peptides from core H2A (HLQLAIR, 453.780 m/z) or the H2AB variant (HLQLAVR). **B**. MS/MS spectra positively identifying the peak at 453.780 m/z as containing the HLQLAIR sequence (core H2A). **C**. MS/MS spectra identifying the peak at 466.772 m/z as containing the HLQLAVR sequence (H2AB). **D**. Quantification of histone variants following contextual conditioning. Values represent average normalized percentage (*n *= 4 per group) of the total for each variant for cage controls (CC) and contextual fear conditioning (FC). Statistical significance was determined using two-tailed t-tests.

To further investigate regulation of histone variants following memory acquisition we performed a quantitative proteomics analysis using a nanoLC-MS/MS platform 1 hour after contextual conditioning. The high similarity of sequence makes it challenging to determine the specificity of the regulation of H2A variants at the protein level using antibody-based technologies. We found that H2AB was detectable in proteomic analyses (Figure 4A) and easily distinguished from other H2A variants. As can be seen in the tandem mass spectra (Figure 4B and 4C) the 14 Da shift produced by the unique presence of a Valine instead of an Isoleucine in H2AB (Additional file 9) allows for the distinction of this novel activity-dependent variant from other H2As using proteomics. Quantification of abundance of histone variants (Figure 4D) demonstrates that H2AB is the most abundant variant in the mouse hippocampus. H2AB is also the only variant significantly downregulated by contextual conditioning (*p *< 0.05), consistent with the microarray and qPCR data, although trends for the reduction of H2A.Z and H2A.X are also observed. Because H2AB has not been previously characterized, we used a molecular evolution approach to investigate its relationship to H2A variants that have been characterized more thoroughly. The resulting multiple sequence alignment and phylogenetic tree (Additional files 9 and 10**S8**) show that H2AB is an H2A variant 100% conserved between human and mouse that is closely related to H2A.X and H2A.J whose role in transcriptional regulation has yet to be studied. Histone variants have emerged as key players regulating epigenetic processes such as chromatin structure and dynamics [52]. Although there is a great deal of knowledge regarding the importance of epigenetic processes such as histone modifications and DNA methylation to memory formation [5-7], the role of histone variants has not yet been explored. It has been previously shown that during rat cortical neuron differentiation there are changes in H2A and H3 variant composition [53]. More recently, an H2A variant (H2BE) necessary for the survival of olfactory neurons was identified, and its expression has been shown to be regulated in an activity-dependent manner [54]. Our data suggests that histone variants, and thus broader epigenetic changes such as global changes in chromatin accessibility, may be an important component of the epigenetic mechanisms recruited at the first sensitive period during memory consolidation.

Memory retrieval had a bigger impact on processes that regulate RNA processing. The downregulation of splicing factor *Rbfox1 *was also replicated by qPCR in an independent cohort (Figure 3C). To our knowledge, this is the first time that *Rbfox1 *has been reported to be regulated by behavior. *Rbfox1 *is an important regulator of both splicing and transcription in brain development [55] that controls neuronal excitability [56]. Clinically, *Rbfox1 *deletion is correlated with developmental delays, learning disabilities and autistic-like features [57]. The results of the functional annotation analysis (Figure 3A), the validation of the down-regulation of Rbfox1 by qPCR (Figure 3C) and the differences in alternative splicing of Per1 observed between FC30' and RT30' (Figure 2B), suggest that post-transcriptional regulation may be of particular importance following memory retrieval. Splicing regulators have been previously reported to change expression during memory formation [58]. However, alternative splicing following memory formation at the genome-wide level has not been previously examined. Greater efforts in future experiments will be directed at understanding the regulation of different spliced isoforms after acquisition and retrieval.

### Memory consolidation and retrieval differentially regulate non-coding RNA expression

We also evaluated the regulation of 1,229 non-coding transcripts with well-established annotation. A summary of non-coding RNAs regulated either at FC30' and RT30' can be found in Additional file 11. MicroRNA genes *miR-212*, *miR-132 *and *miR-219 *were selected for further validation. *MiR-212 *and *miR-132 *are CREB-dependent microRNAs derived from the same precursor that are induced by LTP [59] and play an important role in neuronal plasticity [60]. *MiR-219 *expression is dependent on the activity of NMDA receptors [61], which play an essential role in the acquisition of spatial memories in the hippocampus [62,63]. In addition, we selected three non-coding RNAs whose role in brain function has not been studied previously for further validation: two variants of a small nucleolar RNA (snoRNAs *Snord14d *and *Snord14e*) and *miR-410*, one of the microRNAs contained within the *Mirg *imprinted non-coding RNA cluster. *Snord14e *and *Snord14d *represent some of the highest fold changes seen in our microarray while *Mirg *is strongly expressed in the brain during development [64]. *Mirg *contains at least 13 microRNAs (UCSC genome browser) including *miR-410 *whose expression is known to be specific to the central nervous system [65].

**Figure 5:**
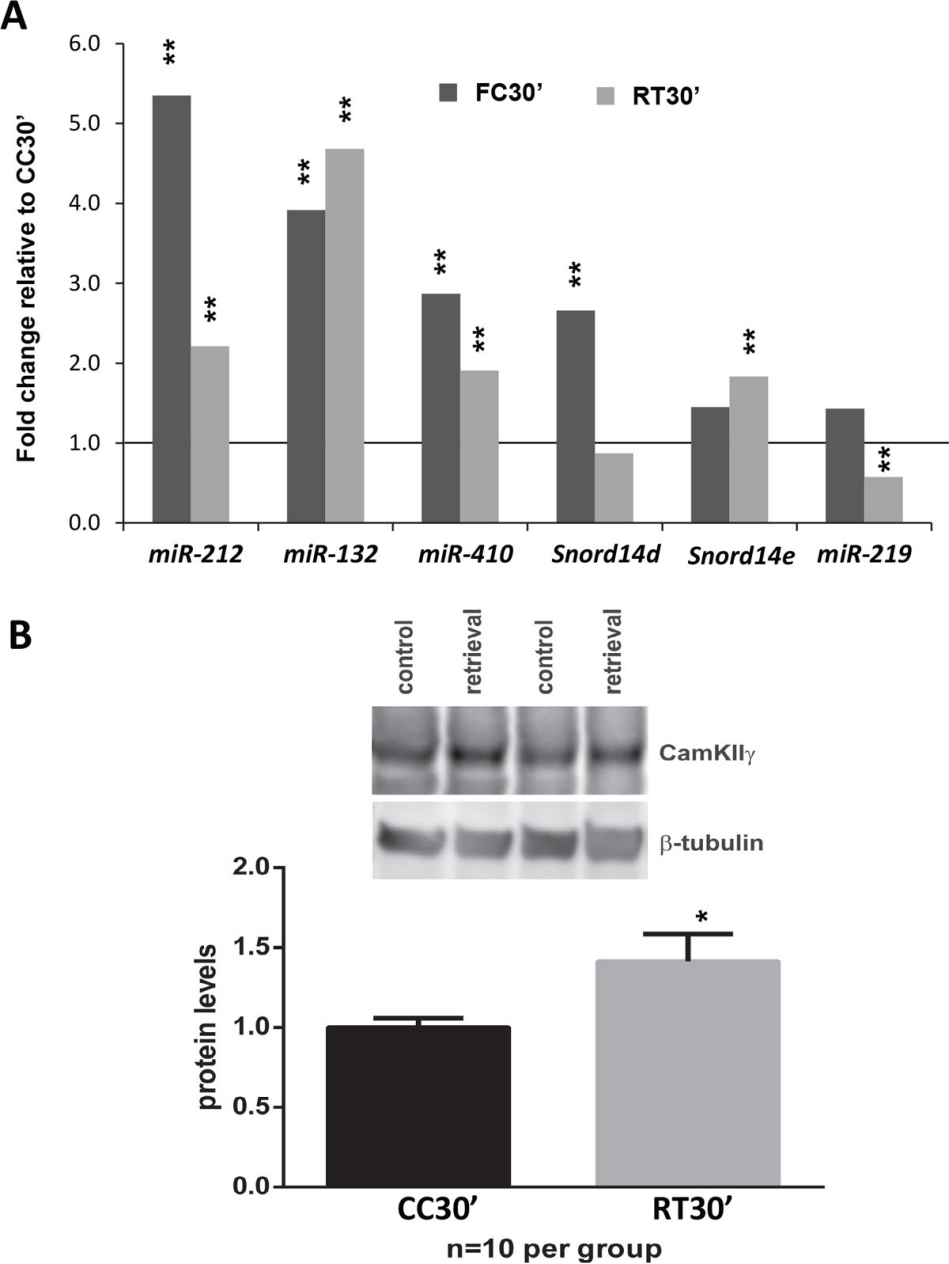
**Non-coding RNA regulation after memory acquisition or retrieval**. **A**. Non-coding RNAs induced at FC30' (dark grey) or RT30' (light gray) by qPCR (*n *= 8). *MiR-219 *is down regulated only at RT30' (box). Expression values from qPCR experiments represent fold changes relative to control (CC30') after normalization against *Snord68 *levels. Statistical significance is highlighted as p-value <0.01 (**) **B**. Protein levels of *miR-219 *target, CAMKIIγ, increase after retrieval, as expected from the downregulation in expression of *miR-219*. Western-blots from hippocampal protein lysates 30 minutes after retrieval (RT30') in an independent cohort of animals (*n *= 10), protein levels are represented as fold change relative to control (CC30'), statistical significance is highlighted as p-value <0.05 (*).

Microarray results may reflect expression of microRNA precursors or processed microRNAs, because RNA hybridized to the microarray was not specifically selected to include small RNA species. Therefore, we performed a second set of experiments isolating small RNAs for subsequent quantification using qPCR to evaluate the expression of mature microRNAs. The results show upregulation of the non-coding RNAs *miR-212*, *miR-132*, *miR-410*, Snord14d and *Snord14e *following memory acquisition (FC30') and retrieval (RT30') (Figure 5A); although the levels of upregulation observed differ between the two time-points. The induction of *miR-212 *and *miR-132 *is not surprising given that they both are induced by LTP [59] and *miR-132 *has been shown to increase in response to the Barnes maze learning paradigm [66]. Transgenic expression of *miR-132 *impairs novel-object recognition memory [67], suggesting that these microRNAs have a functional role in memory storage. Differential regulation of *miR-132 *and *miR-212 *after memory acquisition and retrieval raises the interesting possibility that they target different genes during those processes, although so far the few known experimentally validated targets are shared between *miR-132 *and *miR-212 *[60]. A lot less is known about *miR-410*, but the experience-dependent induction we observe along with the specificity of its expression within the central nervous system and the strong association of one of its targets (MET) with autism [68,69], point to an important role of this microRNA in the regulatory networks that underlie memory consolidation. Brain-specific snoRNAs have been previously reported to be induced in the hippocampus by contextual fear conditioning [70]. However, the function of snoRNAs in post-transcriptional gene regulation in the brain remains largely unexplored.

Downregulation of *miR-219 *was only found to be significant after retrieval (Figure 5A), supporting our previous observation that retrieval downregulates genes involved in RNA processing. *MiR-219 *is known to regulate protein levels of CAMKIIγ [61]. To investigate if the observed reduction of *miR-219 *had a functional effect on protein levels of CAMKIIγ we performed western-blots in an independent cohort of animals (*n *= 10 per group) and found that CAMKIIγ protein levels are indeed significantly increased (*p *< 0.05) after memory retrieval (Figure 5B). It is not clear if the molecular changes we observe after retrieval correspond to reconsolidation or extinction of the memory trace. Based on available literature [71-74], the observed down-regulation of *miR-219 *and up-regulation of CAMKIIγ is consistent with the hypothesis that a single brief re-exposure to the context may inhibit NMDAR activity while maintaining or even increasing CAMKII signaling and thus promote memory reconsolidation while inhibiting extinction. Although it is likely that CAMKIIγ plays an important role in CAMKII mediated signaling, its specific function remains unknown.

## Conclusions

Our study characterizes gene expression genome-wide, both protein coding and non-coding, at several time-points during memory consolidation and following retrieval of memory. We show that training is not the main source of variance in gene expression. We introduce the removal of unwanted variance though normalization to the study of transcriptional changes genome-wide in the context of brain and behavior. Using this approach, we successfully identify novel gene expression changes following contextual conditioning as well as reproduce the majority of the previously reported changes. The largest changes in gene expression related to memory formation are observed 30 minutes after memory acquisition and 30 minutes post retrieval. We identify a novel activity-dependent histone variant, H2AB, and show that is downregulated following memory acquisition. We point to several gene candidates that have not been previously described to be involved in learning and memory, including transcriptional regulators *Sox18*, *Btg2 *and *Sik1*, and splicing factor *Rbfox1*. Finally, we examine genome-wide non-coding RNA regulation following memory acquisition and retrieval, pointing to a likely important role of microRNAs *miR-132*, *miR-212*, *miR-410 *and snoRNAs *Snord14d *and *Snord14e *in posttranscriptional regulation during both processes as well as a specific role for and *miR-219 *and its target CAMKIIγ after retrieval. Epigenetic mechanisms that regulate gene expression have been shown to be essential to long-term memory formation. Our study underlines the importance of two currently understudied epigenetic processes to memory storage and retrieval: histone variants and post-transcriptional RNA regulation; the study of which will expand our knowledge of the molecular mechanisms by which the brain maintains long-lasting changes induced by experience.

## Methods

### Subjects

C57BL/6J adult male mice (2 months of age) were obtained from Jackson Laboratories and housed individually for a week on a 12 hr./12 hr. light/dark schedule with lights on at 7 am (Zeitgeber time (ZT) 0). Food and water were available *ad libitum *throughout the experiment. Each animal was handled daily for 3 days prior to contextual fear conditioning (FC). Handling consisted of manipulation of the animals for 1-2 minutes per mouse in the same room as the experimental setting without exposure to the context. The conditioning protocol entailed a single 2-second, 1.5-mA foot shock, terminating at 2.5 minutes after placement of the mouse in the chamber, starting at 10 am (ZT3) daily. Plexiglass operant chambers (Med-Associates, 31.8 × 25.4 × 26.7 cm) housed in sound-attenuating boxes equipped with individual fans and lights were used for all fear conditioning experiments and controls. The floor consisted of stainless-steel grid rods 3.2 mm in diameter, spaced 0.5 cm apart. Footshock was delivered by a Med-Associates solid-state shock source and grid floor scrambler that delivered a constant current (1.5 mA). Conditioning was quantified by measuring freezing behavior, using automated scoring software (Clever Systems). For microarray experiments, hippocampal dissections were performed immediately following the behavioral treatment, and alternated between FC and control animals. Tissue was collected at 30 minutes (FC30'), 4 hours (FC4), 12 hours (FC12) or 24 hours after FC (FC24) as well as 30 minutes after testing for retrieval of the memory (RT30'). Testing was performed at 24 hours after training over a 5-minute interval, which is sufficient to induce reconsolidation [75,76]. The average freezing was 55 +/- 10%. Tissue was immersed in RNAlater (Qiagen) and immediately frozen. Animals that were handled but not trained were dissected at the same time of day (within 30 minutes) to control for variations due to circadian rhythms (CC30', CC4 and CC12). The protocol was repeated over the course of 2 weeks to obtain 9 animals (2 hippocampi) per group, so that 9 independent FC experiments were represented in each time point and all animals for each group were dissected at the same time of day. For subsequent qPCR and western blots the same protocol was followed, but tissue was collected only at FC30', CC30' and RT30'. Tissue for protein extraction was not immersed in RNAlater. All experiments were approved by the Institution of Animal Care and Use Committee of the University of Pennsylvania and were carried out in accordance with all National Institutes of Health guidelines.

### Microarrays

RNA extraction was performed using Qiagen RNAeasy Microarray Tissue kit. All RNA extractions were performed the same day within a week of tissue collection. Induction of positive controls *Arc*, *Fos *and *Dusp1 *was confirmed by qPCR in the same samples previous to submission to microarrays analysis (data not shown). RNA was submitted to the University of Pennsylvania molecular profiling core for cRNA preparation, hybridization and scanning. Samples were simultaneously hybridized to an Affymetrix Mouse 1.1 Gene-EST 96 sample array plate. Target preparation and hybridization protocols were conducted as described in the Affymetrix GeneChip Expression Analysis Technical Manual. Biotinylated cRNA were prepared from 3 µg total RNA using the Ambion WT Expression Kit. Hybridization, staining and washing was performed using the GeneTitan Hybridization, Wash and Stain Kit for WT Array Plates. Fluorescent signal scanning was performed using the GeneTitan multi-channel instrument. The average signal from two sequential scans was calculated for each microarray feature. RMA normalization was performed using Affymetrix Power tools.

### Statistical analysis

Principal component analysis (PCA) and differential expression analysis was performed using the R programming language and available packages from the R/Bioconductor project [77]. PCA was performed after all expression values were standardized against the mean. The contribution of the first three principal components to the variance is as follows: PC1: 40%, PC2, 17% and PC3, 8%. The data was normalized against the first principal component to remove global systemic artifacts in all subsequent analyses of differential expression. Differential expression was performed against time of day matched controls. Local false discovery rates calculation using empirical null distributions was performed by applying the *locfdr *package to pair-wise t-statistics obtained after PC1 normalization. Estimating an empirical null hypothesis has been shown to add power to estimates of differential gene expression in large-scale studies [38]. However this approach has never been used before to study genome-wide changes gene expression in the brain. Genome-wide fuzzy hierarchical clustering as originally detailed by Gasch and Eisen [78] was implemented using Cluster 3.0 using average linkage on average expression values per condition after RMA normalization. Clustering of individual replicates did not produce any clusters.

### Cross-platform ID mapping, functional annotation and functional interaction analyses

Mapping of gene IDs across different platforms as well as enrichment of functional annotation was assessed using the Database for Visualization and Integrative Discovery (DAVID) [79]. Functional annotation was limited to the following sources to increase information and limit redundancy: GO Biological process, GO Molecular Function, KEGG pathways, and SwissProt and Protein Information Resource keywords. Enrichment for each term was defined relative to the all mouse probe-sets present in the microarray, and was defined as a *p *< 0.05 with at least 3 genes per term per dataset. Fuzzy Heuristical clustering was performed using kappa similarity >0.3 and final group membership of at least 4 functional terms. Enriched functional clusters were defined as enrichment score ≥1.3 (p-value geometric mean between all genes within the cluster <0.05). Functional interaction analysis was performed with STRING [80], using co-expression, experimental, database and PubMed text-mining data. The cut-off interaction score was 0.4 (medium confidence) and the number of additional interactions equal to double the number of initial nodes. Clusters within the interaction network were obtained using K-means [81], K was evaluated from 2-6, results are displayed for the best fitting K value (k = 4).

### Quantitative proteomics

Hippocampi were dissected 1 hour after fear conditioning and flash frozen in liquid nitrogen. Frozen nuclei were thawed on ice and homogenized in 1 mL NIB-250 (15 mM Tric-HCl pH 7.5, 60 mM KCl, 15 mM NaCl, 5 mM MgCl2, 1 mM CaCl2, 250 mM Sucrose, protease inhibitor cocktail (Sigma), 1 mM DTT, 10 mM sodium butyrate) + 0.3% NP-40 in a Type B dounce homogenizer. After 5 minutes on ice, nuclei were pelleted at 600 g for 5 minutes at 4°C and washed in 10:1 NIB-250 without detergent. Nuclei were pelleted and dissolved in 250 ul of 0.4N H2SO4. Acid extraction was performed on a nutator at 4°C for 3 hours, spun at 1500 g for 5 min, and the supernatant was set aside. The pellet was dissolved in another 250 ul of 0.4N H2SO4, rocked for 1 hour at 4°C, and spun at 1500 g for 5 minutes. The two supernatants were combined, 125 ul of 100% TCA was added, and precipitation was allowed to proceed overnight at -20°C. Samples were spun at 3400 g for 10 minutes, aspirated, and washed with 1 mL 0.1% HCl-acetone. Pellets were washed an additional 2× in acetone, allowed to dry, and redissolved in 20 ul H2O. Histone pellets were prepared for mass spectrometry experiments as previously described [82]. In brief, histones were reacted with a 3:1 propionic anhydride/isopropanol mixture, digested with trypsin at a 20:1 protein/trypsin ratio and then reacted with the propionylation reagent one more time to cap the newly generated N-termini. Digested histones were then loaded onto and separated by reversed-phase high-performance liquid chromatography (HPLC) on an EasyLC 1000 system (Thermo, San Jose, CA) using a 75-μm-inner diameter fused silica column packed with 10-15 cm of 5-μm C18 (Michrom, Auburn, CA). The HPLC gradient was a 1-30% buffer B in buffer A (buffer A, 0.1 M acetic acid; buffer B, 95% acetonitrile in 0.1 M acetic acid) for 35 min followed by 30-99% buffer B for 30 min was used to elute peptides, which were ionized into an Orbitrap Velos instrument via electrospray ionization. Peptides were analyzed on The Orbitrap Velos mass spectrometer (ThermoFisher Scientific, San Jose, CA) with full scans of m/z = 290-1200 with a resolution of 30,000, followed by 7 MS/MS spectra collected in the ion trap. All data was manually analyzed and quantified as previously reported [82].

### Ortholog mapping and molecular phylogenetic analysis

Orthologs of mice *Hist2h2ab *were determined using BLAST against the OrthoMCL database [83] (ortholog group OG5_126570). All mouse and human sequences within the ortholog group plus mouse and human H2A.Z were aligned using T-coffee [84]. Phylogenetic reconstruction was performed using PHYML [85] with 100 bootstrap support and displayed using iTOL version 2 [86]. Histone variant macroH2A was not included because the presence of the macro domain produces severe long-branch attraction in the phylogeny reconstruction.

### Quantitative RT-PCR (qPCR)

RNA extraction was performed using Qiagen RNAeasy lipid tissue kit with modifications to obtain both small RNAs as well as mRNAs. Briefly, RNA precipitation was performed using 100% EtOH and washes were performed using Qiagen's RWT buffer. Concentration and purity was quantified by NanoDrop spectrophotometry (Thermo Fisher Scientific, Wilmington, DE). For quantitative real-time RT-PCR, reactions were prepared in 384-well optical reaction plates (ABI, Foster City, CA) with optical adhesive covers (ABI). Two technical replicates were used. Reactions were carried out in ViiA7 real-time PCR system (Invitrogen). For mRNA qPCR, generation of cDNA was carried out by the RETROscript kit (Ambion) with 1 µg of RNA as template. Taqman gene expression assays for all genes were obtained from ABI (Invitrogen). Data was normalized to *Gapdh *prior to calculation of differences.

Relative quantification of gene expression was performed according to ABI's User Bulletin #2. Fold change was calculated using the delta delta Ct method. The data presented is the calculated mean for the biological replicates with *n *= 8 (*i.e*., the number of mice examined). We used t-tests to compare fold change values for each gene in each comparison of interest. Two-tailed p-values are reported. *Fos *induction was used as positive control on all qPCR runs. For microRNA and snoRNAs, qPCR was performed using the miScript system from Qiagen. Reverse transcription was performed using miScript II RT kit using Hiflex buffer. MicroRNA and small nucleolar RNA detection by real-time PCR was performed using the miScript SYBR green PCR Kit. miSCRIPT primer assays were obtained from Qiagen, with the exception of primers for *Snord14d *and *Snord14e*. See Additional data file 12 for assays IDs and sequences. Data was normalized to *Snord68 *prior to calculation of differences. Fold changes were calculated as detailed for mRNAs above.

### Western blot analysis

Frozen hippocampal tissue was homogenized in RIPA buffer with protease and phosphatase inhibitors. Proteins were separated by 4-20% Tris-Glycine SDS-PAGE and transferred to polyvinylidene difluoride (PVDF) membranes. Membranes were blocked in 5% BSA-TBST and incubated overnight at 4°C in primary antibody for CAMKIIγ (1:1000 abcam). They were washed, and incubated with appropriate horseradish peroxidase-conjugated goat anti-mouse or anti-rabbit IgG (1:5000, Santa Cruz) for 1 hour. Blots were exposed using ImageQuant LAS 4000 digital imaging system and quantified using ImageJ. Blots were stripped and re-probed with anti-β-tubulin antibody (1:20000, Sigma). Density of CAMKIIγ signal was normalized to β-tubulin levels.

## Availability of Supporting Data

Microarray data generated in this study is publicly available through GEO (GSE50423). Mass Spec data is available at ChorusProject.org under project name hippocampi histone in mice or with the following links:

https://chorusproject.org/anonymous/download/experiment/8175298673241556154

https://chorusproject.org/anonymous/download/experiment/5303374160982551218

## Competing interests

The authors declare that they have no competing interests.

## Authors' contributions

Study design by L.P., M.W. and T.A. Data collection by L.P., M.W., S.G.P., J.H.K.C and S.L. Data analysis and interpretation by L.P., M.W., S.G.P., J.H.K.C., M.K., N.R.Z., B.A.G., K.P.G. and T.A., manuscript preparation by L.P. with editing by J.H.K.C., M.W., S.G.P, M.K., B.A.G., N.R.Z., K.P.G and T.A.

## Supplementary Material

Additional file 1 **Principal component analysis of genome-wide gene expression changes in mouse hippocampus after conditioning**. Genome-wide gene expression was obtained for 72 samples with *n *= 9 mice/2 hippocampi per group (each collected from an independent experiment) of the following time points: 30 minutes (FC30'), 4 hours (FC4), 12 hours (FC12) and 24 hours after contextual conditioning (FC24) as well as 30 minutes after testing for retrieval of the memory at 24 hours (RT30'). Animals that were handled but not trained were dissected at the same time of day to control for variations due to circadian rhythms (CC30', CC4 and CC12). Samples are color coded in the following order: CC30' (black), FC30' (red), CC4 (green), FC4 (blue), CC12 (light blue), FC12 (pink), FC24 (yellow), RT30' (grey). Loadings for the first, second and third principal components are shown. **A **depicts the loadings of the first principal component, showing high variability in some mice not correlated with sample. **B **depicts the loadings of the second principal component, showing variability from day to day or experiment to experiment not correlated with either day or sample. **C **shows a plot of the scores for PC1 (x-axis) vs. PC2 (y-axis) for all genes in the microarray, showing a very high correlation in a subset of 172 genes for PC1 and PC2 (red oval). **D **depict the loadings of the third principal component, which shows a high correlation between FC4 and CC4, and FC12 and CC12, suggesting that circadian time has a strong influence in gene expression at those time-points regardless of treatment. Click here for file

Additional file 2 **Functional clustering of genes whose scores are correlated between PC1 and PC2**. DAVID functional clustering [87] for the 172 probe-sets (58 genes) whose scores are correlated between PC1 and PC2 (Figure S1C). Enrichment scores (EASE) for functional clusters are calculated as the negative logarithm of the geometric mean of the p-values for individual terms in the cluster including only functional terms with a p-value <0.05 and at least 3 genes and using a cutoff of EASE >1.3 to define enriched clusters (p-value geometric mean <0.05). Only one cluster was identified above the cutoff, with EASE = 3.41, containing 4 functional terms all related to pheromone function. Number of genes that belong to each functional term as well as the term enrichment p-value is displayed on the right. Horizontal bars represent the proportion of the total genes in the list that belong to the individual functional term. Click here for file

Additional file 3 **Genes differentially regulated in the mouse hippocampus due to circadian time**. This table shows the detailed results of comparing gene expression at two times of day: CC30'(10:30 am) and CC12 (10 pm) in the mouse hippocampus. Results are provided at two different fdrs (<0.01, <0.1). Down-regulated probe-sets are highlighted in red, up-regulated ones in green. Click here for file

Additional file 4 **Regulation of circadian gene expression in the hippocampus**. **A **Overlap of genes regulated by time of day in the hippocampus with known circadian oscillators. Transcripts shown to be regulated in the mouse liver at either 24 or 12 hour periods were extracted from Hughes et al [27]. First, probeset ID mapping between Affymetrix mouse Gene Chip 480_2 and Affymetrix Gene 1.1 ST arrays was performed using DAVID [87], allowing all possible mappings. Overlap between the datasets was performed on the basis of the Affymetrix 1.1 Gene ST array IDs. Number of probe sets in each of the datasets is indicated outside of the Venn diagram. **B**. Differential expression over 24 hs of known circadian oscillators (Per1, Per2, Per3) and known plasticity related genes (Arc, Bdnf, Ep300 and Crebbp). Fold change (log2 scale) relative to CC30 (Y-axis) vs. time relative to CC30 (X-axis). Twelve hours' time point (CC12) is highlighted and it overlaps with a peak in activity for mice (10 pm). Click here for file

Additional file 5 **Genes differentially regulated in the mouse hippocampus due to acquisition or retrieval of memory**. This table details the results of comparing gene expression at all-time points after contextual conditioning (FC30', FC4, FC12 and RT30') with their respective circadian time controls (CC30', CC4 and CC12). The overlap between genes regulated at FC30 and RT30 is also provided. Results are shown at two different false discovery rates (fdrs <0.01, <0.1). FC24 is not displayed because no gene expression changes were detected at either fdr. Table provided as Excel spreadsheet. Down-regulated probe-sets are highlighted in red, up-regulated ones in green. Average expression for each probe-set at each time-point is provided (log2 scale). Genes that had been validated to be differentially expressed after contextual conditioning in other studies are highlighted in orange; genes validated in this study are highlighted in yellow. Click here for file

Additional file 6 **Functional clustering of genes upregulated at FC30' and RT30'**. DAVID functional clustering [87] for the 27 genes that are up-regulated at FC30' and RT30' at an fdr<0.1. Enrichment scores (EASE) for functional clusters are calculated as the negative logarithm of the geometric mean of the enrichment p-values for individual functional terms in the cluster. Only terms with p-value <0.05 with and at least 3 genes are included in the clustering. Only clusters with EASE >1.3 are considered enriched clusters (p-value geometric mean <0.05). Only one cluster with enrichment score of 2.63, containing 30 functional terms was identified. All functional terms are related to regulation of transcription. Number of genes in each cluster as well as individual enrichment p-values for each functional term is displayed. Click here for file

Additional file 7 **Functional clustering of genes down-regulated at FC30' and RT30'**. DAVID functional clustering [87] for genes that are down-regulated at FC30' or RT30' (fdr<0.1). Enrichment scores (EASE) for functional clusters are calculated as the negative logarithm of the geometric mean of the enrichment p-values for individual functional terms in the cluster. Only terms with p-value <0.05 with and at least 3 genes are included in the clustering. Only clusters with EASE >1.3 are considered enriched clusters (p-value geometric mean <0.05). **A**. Functional clustering for the set of 63 genes (72 probe-sets) downregulated at FC30' but not at RT30'. Only one cluster with enrichment score of 2.73, containing 20 functional terms was identified. All functional terms are related to chromatin assembly. **B **Functional clustering for the set of 84 genes (107 probe-sets) downregulated at RT30' but not at FC30'. Only one cluster with enrichment score of 1.58, containing 6 functional terms was identified. All functional terms are related to RNA processing. Number of genes that belong to each functional term as well as the term enrichment p-value is displayed on the right. Horizontal bars represent the proportion of the total genes in the list that belong to the individual functional term. Click here for file

Additional file 8 **Regulatory networks induced by memory acquisition and retrieval**. Functional interaction analysis of protein coding genes induced at FC30' and RT30' (STRING [80]). **A**. Interaction network of genes induced at FC30' and RT30'. Only 20 out of the 27 non-intronic probesets up-regulated at FC30' and RT30' (fdr <0.1) mapped to mouse proteins present in the STRING database (colored nodes), 40 additional nodes were incorporated based on predicted interactions (white nodes). Interactions between nodes are represented as colored lines, only medium to high confidence interactions are shown (interaction score > 0.4). Colors represent sources of information as follows: co-expression (black), experiments (magenta), databases (blue) and PubMed text-mining (green). Interaction score > 0.4 for all shown interactions. **B **Results of K-mean clustering (k = 4) of the interaction network shown above. Clusters are color coded, red nodes representing unclustered nodes. Three clear clusters are present in the network: a MAPK/CREB cluster (yellow), an Nf-κB cluster (in green) and a Per1 cluster (in blue). Interactions within clusters are represented by solid colored lines, while interactions among clusters are represented by dashed colored lines. Colors represent sources of information as previously described. Click here for file

Additional file 9 **Multiple sequence alignment of the c-terminal portion of Histone 2A**. Mouse Hist2h2ab (H2AB) was mapped to orholog group OG5_126570 using OrthoMCL [83]. Human and mouse Ensembl sequences from the group were aligned using T-coffee [88]. Positions 69-145 are displayed, since no differences between H2AB and other H2A sequences are observed in the N-terminus. Gene names are displayed, m depicts mouse sequences, h depicts human sequences. Residues conserved in all sequences are not-color coded. H2AB and the residues unique to this variant are highlighted in green. Magenta: AA residues for which only one histone variant differs from others. Orange-yellow shades: AA residues for which several H2A sequences differ from each other. The red box highlights the peptide used to identify H2AB in the quantitative proteomics analysis. Click here for file

Additional file 10 **Molecular Phylogeny of human and mouse orthologs of H2AB (Hist2h2ab)**. Mouse Hist2h2ab was mapped to orholog group OG5_126570 using OrthoMCL [83]. Human and mouse Ensembl sequences from the group were aligned using T-coffee [88] and phylogeny reconstruction was performed using PhyML [85] using aLRT likelihood to calculate branch support. Gene names are displayed, m depicts mouse sequences, h depicts human sequences. Black dots indicate braches with >0.8 support. Click here for file

Additional file 11 **MicroRNAs regulated after acquisition or retrieval of memory in our microarray study**. Non-coding RNAs (precursors) regulated at FC30' or RT30' in the microarray study. Values represent fold change relative to control (CC30'). Green background: up-regulated, Red background: down-regulated. Statistical significance is highlighted by font color: fdr <0.01 (in red) or fdr <0.1 (in orange). N.S: not significant differences in gene expression. Click here for file

Additional file 12 **Assays IDs or primer sequences for genes tested by qPCR**. Click here for file

## Declarations

Cost of article publications fees was covered through grant R01 MH087463 from the NIH/NIMH.

This article has been published as part of *BMC Genomics *Volume 16 Supplement 5, 2015: Proceedings of the 10th International Conference of the Brazilian Association for Bioinformatics and Computational Biology (X-Meeting 2014). The full contents of the supplement are available online at http://www.biomedcentral.com/bmcgenomics/supplements/16/S5.

## References

[B1] Scherer A Batch effects and noise in microarray experiments: sources and solutions 2009 John Wiley & Sons

[B2] Irizarry RA Hobbs B Collin F Beazer-Barclay YD Antonellis KJ Scherf U Speed TP Exploration, normalization, and summaries of high density oligonucleotide array probe level data Biostatistics 2003 4 249 264 10.1093/biostatistics/4.2.249 12925520

[B3] Agranoff BW Davis RE Casola L Lim R Actinomycin D blocks formation of memory of shock-avoidance in goldfish Science 1967 158 1600 1601 10.1126/science.158.3808.1600 6060370

[B4] Flood JF Rosenzweig MR Bennett EL Orme AE The influence of duration of protein synthesis inhibition on memory Physiology & behavior 1973 10 555 562 10.1016/0031-9384(73)90221-7 4736141

[B5] Zovkic IB Guzman-Karlsson MC Sweatt JD Epigenetic regulation of memory formation and maintenance Learn Mem 2013 20 61 74 10.1101/lm.026575.112 23322554 PMC3549063

[B6] Peixoto L Abel T The role of histone acetylation in memory formation and cognitive impairments Neuropsychopharmacology 2013 38 62 76 10.1038/npp.2012.86 22669172 PMC3521994

[B7] Nelson ED Monteggia LM Epigenetics in the mature mammalian brain: effects on behavior and synaptic transmission Neurobiol Learn Mem 2011 96 53 60 10.1016/j.nlm.2011.02.015 21396474 PMC3463371

[B8] Alberini CM Transcription factors in long-term memory and synaptic plasticity Physiol Rev 2009 89 121 145 10.1152/physrev.00017.2008 19126756 PMC3883056

[B9] Bourtchouladze R Abel T Berman N Gordon R Lapidus K Kandel ER Different training procedures recruit either one or two critical periods for contextual memory consolidation, each of which requires protein synthesis and PKA Learn Mem 1998 5 365 374 10454361 PMC311273

[B10] Igaz LM Vianna MR Medina JH Izquierdo I Two time periods of hippocampal mRNA synthesis are required for memory consolidation of fear-motivated learning J Neurosci 2002 22 6781 6789 12151558 10.1523/JNEUROSCI.22-15-06781.2002 PMC6758123

[B11] Bekinschtein P Katche C Slipczuk L Gonzalez C Dorman G Cammarota M Izquierdo I Medina JH Persistence of Long-Term Memory Storage: New Insights into its Molecular Signatures in the Hippocampus and Related Structures Neurotox Res10.1007/s12640-010-9155-5 20151243

[B12] Vianna MR Igaz LM Coitinho AS Medina JH Izquierdo I Memory extinction requires gene expression in rat hippocampus Neurobiol Learn Mem 2003 79 199 203 10.1016/S1074-7427(03)00003-0 12676518

[B13] Power AE Berlau DJ McGaugh JL Steward O Anisomycin infused into the hippocampus fails to block "reconsolidation" but impairs extinction: the role of re-exposure duration Learn Mem 2006 13 27 34 10.1101/lm.91206 16452651 PMC1360130

[B14] Mamiya N Fukushima H Suzuki A Matsuyama Z Homma S Frankland PW Kida S Brain region-specific gene expression activation required for reconsolidation and extinction of contextual fear memory J Neurosci 2009 29 402 413 10.1523/JNEUROSCI.4639-08.2009 19144840 PMC6664934

[B15] Motanis H Maroun M Differential involvement of protein synthesis and actin rearrangement in the reacquisition of contextual fear conditioning Hippocampus 2012 22 494 500 10.1002/hipo.20915 21240917

[B16] Hermey G Mahlke C Gutzmann JJ Schreiber J Bluthgen N Kuhl D Genome-wide profiling of the activity-dependent hippocampal transcriptome PLoS One 2013 8 e76903 10.1371/journal.pone.0076903 24146943 PMC3798291

[B17] Keeley MB Wood MA Isiegas C Stein J Hellman K Hannenhalli S Abel T Differential transcriptional response to nonassociative and associative components of classical fear conditioning in the amygdala and hippocampus Learning & memory 2006 13 135 142 10.1101/lm.86906 16547164 PMC1409829

[B18] Levenson JM Choi S Lee SY Cao YA Ahn HJ Worley KC Pizzi M Liou HC Sweatt JD A bioinformatics analysis of memory consolidation reveals involvement of the transcription factor c-rel The Journal of neuroscience : the official journal of the Society for Neuroscience 2004 24 3933 3943 10.1523/JNEUROSCI.5646-03.2004 15102909 PMC6729420

[B19] Barnes P Kirtley A Thomas KL Quantitatively and qualitatively different cellular processes are engaged in CA1 during the consolidation and reconsolidation of contextual fear memory Hippocampus 2012 22 149 171 10.1002/hipo.20879 21080409

[B20] Griggs EM Young EJ Rumbaugh G Miller CA MicroRNA-182 regulates amygdala-dependent memory formation J Neurosci 2013 33 1734 1740 10.1523/JNEUROSCI.2873-12.2013 23345246 PMC3711533

[B21] Ahn HJ Hernandez CM Levenson JM Lubin FD Liou HC Sweatt JD c-Rel, an NF-kappaB family transcription factor, is required for hippocampal long-term synaptic plasticity and memory formation Learn Mem 2008 15 539 549 10.1101/lm.866408 18626097 PMC2505322

[B22] Leach PT Poplawski SG Kenney JW Hoffman B Liebermann DA Abel T Gould TJ Gadd45b knockout mice exhibit selective deficits in hippocampus-dependent long-term memory Learn Mem 2012 19 319 324 10.1101/lm.024984.111 22802593 PMC3407936

[B23] Abdi H Williams LJ Principal component analysis Wiley Interdisciplinary Reviews: Computational Statistics 2010 2 433 459 10.1002/wics.101

[B24] Devan BD Goad EH Petri HL Antoniadis EA Hong NS Ko CH Leblanc L Lebovic SS Lo Q Ralph MR McDonald RJ Circadian phase-shifted rats show normal acquisition but impaired long-term retention of place information in the water task Neurobiology of learning and memory 2001 75 51 62 10.1006/nlme.1999.3957 11124046

[B25] Tapp WN Holloway FA Phase shifting circadian rhythms produces retrograde amnesia Science 1981 211 1056 1058 10.1126/science.7193351 7193351

[B26] Stephan FK Kovacevic NS Multiple retention deficit in passive avoidance in rats is eliminated by suprachiasmatic lesions Behavioral biology 1978 22 456 462 10.1016/S0091-6773(78)92565-8 567972

[B27] Hughes ME DiTacchio L Hayes KR Vollmers C Pulivarthy S Baggs JE Panda S Hogenesch JB Harmonics of circadian gene transcription in mammals PLoS genetics 2009 5 e1000442 10.1371/journal.pgen.1000442 19343201 PMC2654964

[B28] Su AI Wiltshire T Batalov S Lapp H Ching KA Block D Zhang J Soden R Hayakawa M Kreiman G A gene atlas of the mouse and human protein-encoding transcriptomes Proceedings of the National Academy of Sciences of the United States of America 2004 101 6062 6067 10.1073/pnas.0400782101 15075390 PMC395923

[B29] Steward O Wallace CS Lyford GL Worley PF Synaptic activation causes the mRNA for the IEG Arc to localize selectively near activated postsynaptic sites on dendrites Neuron 1998 21 741 751 10.1016/S0896-6273(00)80591-7 9808461

[B30] Zheng F Zhou X Moon C Wang H Regulation of brain-derived neurotrophic factor expression in neurons International journal of physiology, pathophysiology and pharmacology 2012 4 188 200 PMC3544221 23320132

[B31] Oike Y Hata A Mamiya T Kaname T Noda Y Suzuki M Yasue H Nabeshima T Araki K Yamamura K Truncated CBP protein leads to classical Rubinstein-Taybi syndrome phenotypes in mice: implications for a dominant-negative mechanism Human molecular genetics 1999 8 387 396 10.1093/hmg/8.3.387 9949198

[B32] Wood MA Kaplan MP Park A Blanchard EJ Oliveira AM Lombardi TL Abel T Transgenic mice expressing a truncated form of CREB-binding protein (CBP) exhibit deficits in hippocampal synaptic plasticity and memory storage Learning & memory 2005 12 111 119 10.1101/lm.86605 15805310 PMC1074328

[B33] Alarcon JM Malleret G Touzani K Vronskaya S Ishii S Kandel ER Barco A Chromatin acetylation, memory, and LTP are impaired in CBP+/- mice: a model for the cognitive deficit in Rubinstein-Taybi syndrome and its amelioration Neuron 2004 42 947 959 10.1016/j.neuron.2004.05.021 15207239

[B34] Korzus E Rosenfeld MG Mayford M CBP histone acetyltransferase activity is a critical component of memory consolidation Neuron 2004 42 961 972 10.1016/j.neuron.2004.06.002 15207240 PMC8048715

[B35] Bourtchouladze R Lidge R Catapano R Stanley J Gossweiler S Romashko D Scott R Tully T A mouse model of Rubinstein-Taybi syndrome: defective long-term memory is ameliorated by inhibitors of phosphodiesterase 4 Proceedings of the National Academy of Sciences of the United States of America 2003 100 10518 10522 10.1073/pnas.1834280100 12930888 PMC193593

[B36] Oliveira AM Estevez MA Hawk JD Grimes S Brindle PK Abel T Subregion-specific p300 conditional knock-out mice exhibit long-term memory impairments Learning & memory 2011 18 161 169 10.1101/lm.1939811 21345974 PMC3056518

[B37] Oliveira AM Wood MA McDonough CB Abel T Transgenic mice expressing an inhibitory truncated form of p300 exhibit long-term memory deficits Learning & memory 2007 14 564 572 10.1101/lm.656907 17761541 PMC1994075

[B38] Efron B Large-scale simultaneous hypothesis testing: The choice of a null hypothesis Journal of the American Statistical Association 2004 99 96 104 10.1198/016214504000000089

[B39] Benjamini Y Hochberg Y Controlling the False Discovery Rate - a Practical and Powerful Approach to Multiple Testing Journal of the Royal Statistical Society Series B-Methodological 1995 57 289 300

[B40] Farioli-Vecchioli S Saraulli D Costanzi M Leonardi L Cina I Micheli L Nutini M Longone P Oh SP Cestari V Tirone F Impaired terminal differentiation of hippocampal granule neurons and defective contextual memory in PC3/Tis21 knockout mice PLoS One 2009 4 e8339 10.1371/journal.pone.0008339 20020054 PMC2791842

[B41] Li H Radford JC Ragusa MJ Shea KL McKercher SR Zaremba JD Soussou W Nie Z Kang YJ Nakanishi N Transcription factor MEF2C influences neural stem/progenitor cell differentiation and maturation in vivo Proc Natl Acad Sci USA 2008 105 9397 9402 10.1073/pnas.0802876105 18599437 PMC2453715

[B42] Cole CJ Mercaldo V Restivo L Yiu AP Sekeres MJ Han JH Vetere G Pekar T Ross PJ Neve RL MEF2 negatively regulates learning-induced structural plasticity and memory formation Nat Neurosci 2012 15 1255 1264 10.1038/nn.3189 22885849

[B43] Berdeaux R Goebel N Banaszynski L Takemori H Wandless T Shelton GD Montminy M SIK1 is a class II HDAC kinase that promotes survival of skeletal myocytes Nat Med 2007 13 597 603 10.1038/nm1573 17468767

[B44] Finsterwald C Carrard A Martin JL Role of salt-inducible kinase 1 in the activation of MEF2-dependent transcription by BDNF PLoS One 2013 8 e54545 10.1371/journal.pone.0054545 23349925 PMC3551851

[B45] Li S Zhang C Takemori H Zhou Y Xiong ZQ TORC1 regulates activity-dependent CREB-target gene transcription and dendritic growth of developing cortical neurons J Neurosci 2009 29 2334 2343 10.1523/JNEUROSCI.2296-08.2009 19244510 PMC6666266

[B46] Katoh Y Takemori H Lin XZ Tamura M Muraoka M Satoh T Tsuchiya Y Min L Doi J Miyauchi A Silencing the constitutive active transcription factor CREB by the LKB1-SIK signaling cascade FEBS J 2006 273 2730 2748 10.1111/j.1742-4658.2006.05291.x 16817901

[B47] Jilg A Lesny S Peruzki N Schwegler H Selbach O Dehghani F Stehle JH Temporal dynamics of mouse hippocampal clock gene expression support memory processing Hippocampus 2010 20 377 388 19437502 10.1002/hipo.20637

[B48] Cheval H Chagneau C Levasseur G Veyrac A Faucon-Biguet N Laroche S Davis S Distinctive features of Egr transcription factor regulation and DNA binding activity in CA1 of the hippocampus in synaptic plasticity and consolidation and reconsolidation of fear memory Hippocampus 2012 22 631 642 10.1002/hipo.20926 21425206

[B49] Lubin FD Roth TL Sweatt JD Epigenetic regulation of BDNF gene transcription in the consolidation of fear memory J Neurosci 2008 28 10576 10586 10.1523/JNEUROSCI.1786-08.2008 18923034 PMC3312036

[B50] Sakamoto K Karelina K Obrietan K CREB: a multifaceted regulator of neuronal plasticity and protection Journal of neurochemistry 2011 116 1 9 10.1111/j.1471-4159.2010.07080.x 21044077 PMC3575743

[B51] Oikawa K Odero GL Platt E Neuendorff M Hatherell A Bernstein MJ Albensi BC NF-kappaB p50 subunit knockout impairs late LTP and alters long term memory in the mouse hippocampus BMC neuroscience 2012 13 45 10.1186/1471-2202-13-45 22553912 PMC3394209

[B52] Chen P Zhao J Li G Histone variants in development and diseases J Genet Genomics 2013 40 355 365 10.1016/j.jgg.2013.05.001 23876776

[B53] Pina B Suau P Changes in histones H2A and H3 variant composition in differentiating and mature rat brain cortical neurons Dev Biol 1987 123 51 58 10.1016/0012-1606(87)90426-X 3622934

[B54] Santoro SW Dulac C The activity-dependent histone variant H2BE modulates the life span of olfactory neurons Elife 2012 1 e00070 23240083 10.7554/eLife.00070 PMC3510456

[B55] Fogel BL Wexler E Wahnich A Friedrich T Vijayendran C Gao F Parikshak N Konopka G Geschwind DH RBFOX1 regulates both splicing and transcriptional networks in human neuronal development Hum Mol Genet 2012 21 4171 4186 10.1093/hmg/dds240 22730494 PMC3441119

[B56] Gehman LT Stoilov P Maguire J Damianov A Lin CH Shiue L Ares M Mody I Black DL The splicing regulator Rbfox1 (A2BP1) controls neuronal excitation in the mammalian brain Nat Genet 2011 43 706 711 10.1038/ng.841 21623373 PMC3125461

[B57] Mikhail FM Lose EJ Robin NH Descartes MD Rutledge KD Rutledge SL Korf BR Carroll AJ Clinically relevant single gene or intragenic deletions encompassing critical neurodevelopmental genes in patients with developmental delay, mental retardation, and/or autism spectrum disorders Am J Med Genet A 2011 155A 2386 2396 22031302 10.1002/ajmg.a.34177

[B58] Antunes-Martins A Mizuno K Irvine EE Lepicard EM Giese KP Sex-dependent up-regulation of two splicing factors, Psf and Srp20, during hippocampal memory formation Learn Mem 2007 14 693 702 10.1101/lm.640307 17911373 PMC2044560

[B59] Wibrand K Panja D Tiron A Ofte ML Skaftnesmo KO Lee CS Pena JT Tuschl T Bramham CR Differential regulation of mature and precursor microRNA expression by NMDA and metabotropic glutamate receptor activation during LTP in the adult dentate gyrus in vivo Eur J Neurosci 2010 31 636 645 10.1111/j.1460-9568.2010.07112.x 20384810 PMC3791877

[B60] Tognini P Pizzorusso T MicroRNA212/132 family: molecular transducer of neuronal function and plasticity Int J Biochem Cell Biol 2012 44 6 10 10.1016/j.biocel.2011.10.015 22062950

[B61] Kocerha J Faghihi MA Lopez-Toledano MA Huang J Ramsey AJ Caron MG Sales N Willoughby D Elmen J Hansen HF MicroRNA-219 modulates NMDA receptor-mediated neurobehavioral dysfunction Proc Natl Acad Sci USA 2009 106 3507 3512 10.1073/pnas.0805854106 19196972 PMC2651305

[B62] Tsien JZ Huerta PT Tonegawa S The essential role of hippocampal CA1 NMDA receptor-dependent synaptic plasticity in spatial memory Cell 1996 87 1327 1338 10.1016/S0092-8674(00)81827-9 8980238

[B63] Nakazawa K McHugh TJ Wilson MA Tonegawa S NMDA receptors, place cells and hippocampal spatial memory Nat Rev Neurosci 2004 5 361 372 10.1038/nrn1385 15100719

[B64] Han Z He H Zhang F Huang Z Liu Z Jiang H Wu Q Spatiotemporal expression pattern of Mirg, an imprinted non-coding gene, during mouse embryogenesis J Mol Histol 2012 43 1 8 10.1007/s10735-011-9367-x 22033866

[B65] Wheeler G Ntounia-Fousara S Granda B Rathjen T Dalmay T Identification of new central nervous system specific mouse microRNAs FEBS Lett 2006 580 2195 2200 10.1016/j.febslet.2006.03.019 16566924

[B66] Hansen KF Karelina K Sakamoto K Wayman GA Impey S Obrietan K miRNA-132: a dynamic regulator of cognitive capacity Brain Struct Funct 2013 218 817 831 10.1007/s00429-012-0431-4 22706759 PMC3508255

[B67] Hansen KF Sakamoto K Wayman GA Impey S Obrietan K Transgenic miR132 alters neuronal spine density and impairs novel object recognition memory PLoS One 2010 5 e15497 10.1371/journal.pone.0015497 21124738 PMC2993964

[B68] Chen L Zhang J Feng Y Li R Sun X Du W Piao X Wang H Yang D Sun Y MiR-410 regulates MET to influence the proliferation and invasion of glioma Int J Biochem Cell Biol 2012 44 1711 1717 10.1016/j.biocel.2012.06.027 22750473

[B69] Campbell DB D'Oronzio R Garbett K Ebert PJ Mirnics K Levitt P Persico AM Disruption of cerebral cortex MET signaling in autism spectrum disorder Ann Neurol 2007 62 243 250 10.1002/ana.21180 17696172

[B70] Rogelj B Hartmann CE Yeo CH Hunt SP Giese KP Contextual fear conditioning regulates the expression of brain-specific small nucleolar RNAs in hippocampus Eur J Neurosci 2003 18 3089 3096 10.1111/j.1460-9568.2003.03026.x 14656304

[B71] Da Silva WC Cardoso G Bonini JS Benetti F Izquierdo I Memory reconsolidation and its maintenance depend on L-voltage-dependent calcium channels and CaMKII functions regulating protein turnover in the hippocampus Proc Natl Acad Sci USA 2013 110 6566 6570 10.1073/pnas.1302356110 23576750 PMC3631664

[B72] Fiorenza NG Rosa J Izquierdo I Myskiw JC Modulation of the extinction of two different fear-motivated tasks in three distinct brain areas Behav Brain Res 2012 232 210 216 10.1016/j.bbr.2012.04.015 22525015

[B73] Myers KM Carlezon WA Davis M Glutamate receptors in extinction and extinction-based therapies for psychiatric illness Neuropsychopharmacology 2011 36 274 293 10.1038/npp.2010.88 20631689 PMC2994960

[B74] Sanhueza M Lisman J The CaMKII/NMDAR complex as a molecular memory Molecular Brain 2013 6 10 10.1186/1756-6606-6-10 23410178 PMC3582596

[B75] Suzuki A Josselyn SA Frankland PW Masushige S Silva AJ Kida S Memory reconsolidation and extinction have distinct temporal and biochemical signatures J Neurosci 2004 24 4787 4795 10.1523/JNEUROSCI.5491-03.2004 15152039 PMC6729467

[B76] von Hertzen LS Giese KP Memory reconsolidation engages only a subset of immediate-early genes induced during consolidation J Neurosci 2005 25 1935 1942 10.1523/JNEUROSCI.4707-04.2005 15728833 PMC6726052

[B77] Gentleman RC Carey VJ Bates DM Bolstad B Dettling M Dudoit S Ellis B Gautier L Ge Y Gentry J Bioconductor: open software development for computational biology and bioinformatics Genome Biol 2004 5 R80 10.1186/gb-2004-5-10-r80 15461798 PMC545600

[B78] Gasch AP Eisen MB Exploring the conditional coregulation of yeast gene expression through fuzzy k-means clustering Genome Biology 2002 3 RESEARCH0059 12429058 10.1186/gb-2002-3-11-research0059 PMC133443

[B79] Dennis G Sherman BT Hosack DA Yang J Gao W Lane HC Lempicki RA DAVID: Database for Annotation, Visualization, and Integrated Discovery Genome Biol 2003 4 P3 10.1186/gb-2003-4-5-p3 12734009 PMC193660

[B80] Franceschini A Szklarczyk D Frankild S Kuhn M Simonovic M Roth A Lin J Minguez P Bork P von Mering C Jensen LJ STRING v9.1: protein-protein interaction networks, with increased coverage and integration Nucleic Acids Research 2013 41 D808 815 10.1093/nar/gks1094 23203871 PMC3531103

[B81] Hartigan JA Wong MA Algorithm AS 136: A K-Means Clustering Algorithm Journal of the Royal Statistical Society Series C (Applied Statistics) 1979 28 100 108

[B82] Plazas-Mayorca MD Zee BM Young NL Fingerman IM LeRoy G Briggs SD Garcia BA One-pot shotgun quantitative mass spectrometry characterization of histones J Proteome Res 2009 8 5367 5374 10.1021/pr900777e 19764812 PMC2798817

[B83] Fischer S Brunk BP Chen F Gao X Harb OS Iodice JB Shanmugam D Roos DS Stoeckert CJ Jr Using OrthoMCL to assign proteins to OrthoMCL-DB groups or to cluster proteomes into new ortholog groups Curr Protoc Bioinformatics 2011 Chapter 6 Unit 6 12 11 19 21901743 10.1002/0471250953.bi0612s35 PMC3196566

[B84] Taly JF Magis C Bussotti G Chang JM Di Tommaso P Erb I Espinosa-Carrasco J Kemena C Notredame C Using the T-Coffee package to build multiple sequence alignments of protein, RNA, DNA sequences and 3D structures Nat Protoc 2011 6 1669 1682 10.1038/nprot.2011.393 21979275

[B85] Criscuolo A morePhyML: improving the phylogenetic tree space exploration with PhyML 3 Mol Phylogenet Evol 2011 61 944 948 10.1016/j.ympev.2011.08.029 21925283

[B86] Letunic I Bork P Interactive Tree Of Life v2: online annotation and display of phylogenetic trees made easy Nucleic Acids Res 2011 39 W475 478 10.1093/nar/gkr201 21470960 PMC3125724

[B87] Jiao XL Sherman BT Huang DW Stephens R Baseler MW Lane HC Lempicki RA DAVID-WS: a stateful web service to facilitate gene/protein list analysis Bioinformatics 2012 28 1805 1806 10.1093/bioinformatics/bts251 22543366 PMC3381967

[B88] Di Tommaso P Moretti S Xenarios I Orobitg M Montanyola A Chang JM Taly JF Notredame C T-Coffee: a web server for the multiple sequence alignment of protein and RNA sequences using structural information and homology extension Nucleic Acids Res 2011 39 W13 17 10.1093/nar/gkr245 21558174 PMC3125728

