# Supplementary figures and images for: Memory acquisition and retrieval impact different epigenetic processes that regulate gene expression
Source: BMC Genomics. 2015 May 26;16(Suppl 5):S5. doi: 10.1186/1471-2164-16-S5-S5 (PMC4460846; doi:10.1186/1471-2164-16-S5-S5)

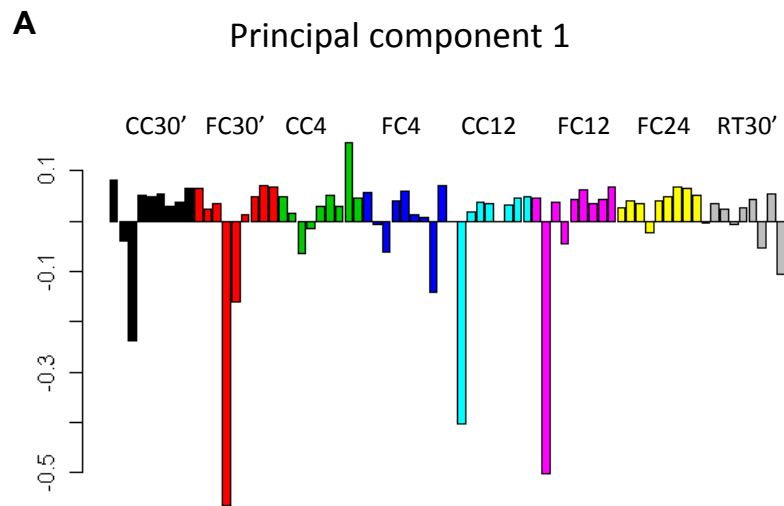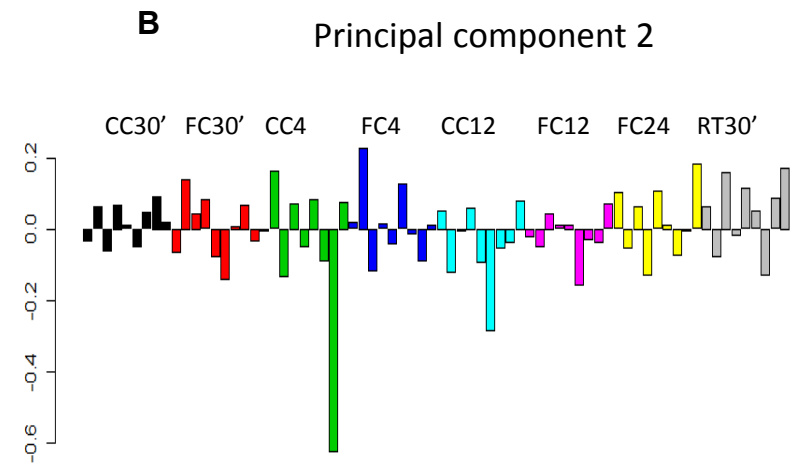

**C** Correlation of gene contributions

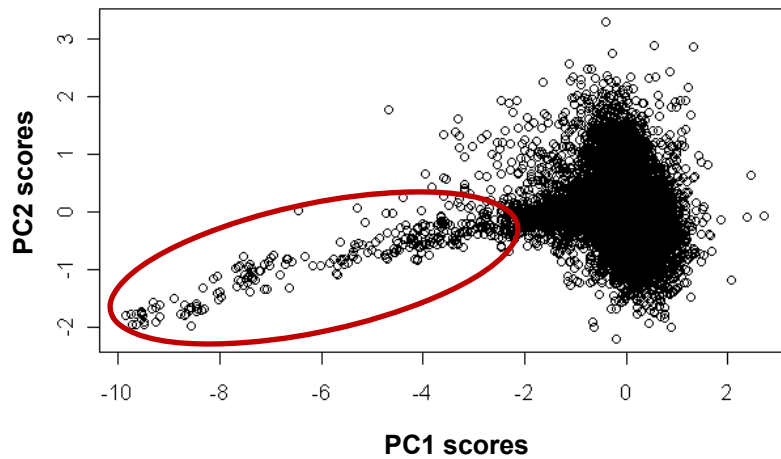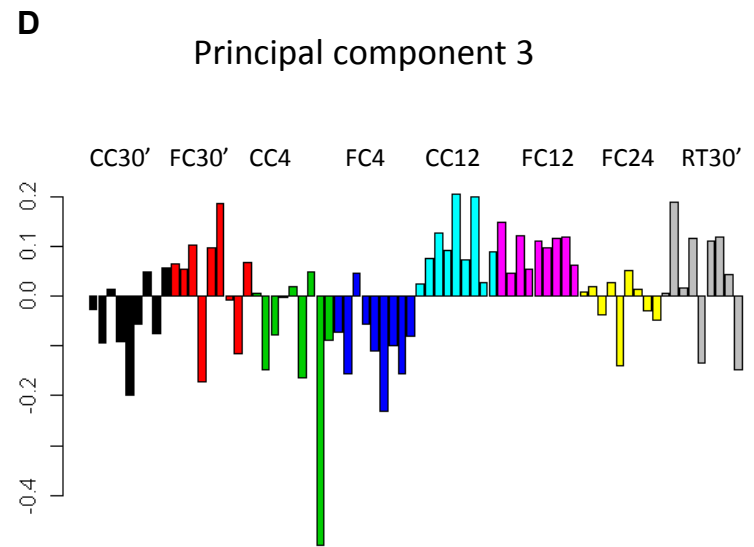

Supplement: Additional file 1 — Principal component analysis of genome-wide gene expression changes in mouse hippocampus after conditioning. Genome-wide gene expression was obtained for 72 samples with n = 9 mice/2 hippocampi per group (each collected from an independent experiment) of the following time points: 30 minutes (FC30'), 4 hours (FC4), 12 hours (FC12) and 24 hours after contextual conditioning (FC24) as well as 30 minutes after testing for retrieval of the memory at 24 hours (RT30'). Animals that were handled but not trained were dissected at the same time of day to control for variations due to circadian rhythms (CC30', CC4 and CC12). Samples are color coded in the following order: CC30' (black), FC30' (red), CC4 (green), FC4 (blue), CC12 (light blue), FC12 (pink), FC24 (yellow), RT30' (grey). Loadings for the first, second and third principal components are shown. A depicts the loadings of the first principal component, showing high variability in some mice not correlated with sample. B depicts the loadings of the second principal component, showing variability from day to day or experiment to experiment not correlated with either day or sample. C shows a plot of the scores for PC1 (x-axis) vs. PC2 (y-axis) for all genes in the microarray, showing a very high correlation in a subset of 172 genes for PC1 and PC2 (red oval). D depict the loadings of the third principal component, which shows a high correlation between FC4 and CC4, and FC12 and CC12, suggesting that circadian time has a strong influence in gene expression at those time-points regardless of treatment. [file 1471-2164-16-S5-S5-S1.pdf]

S3A

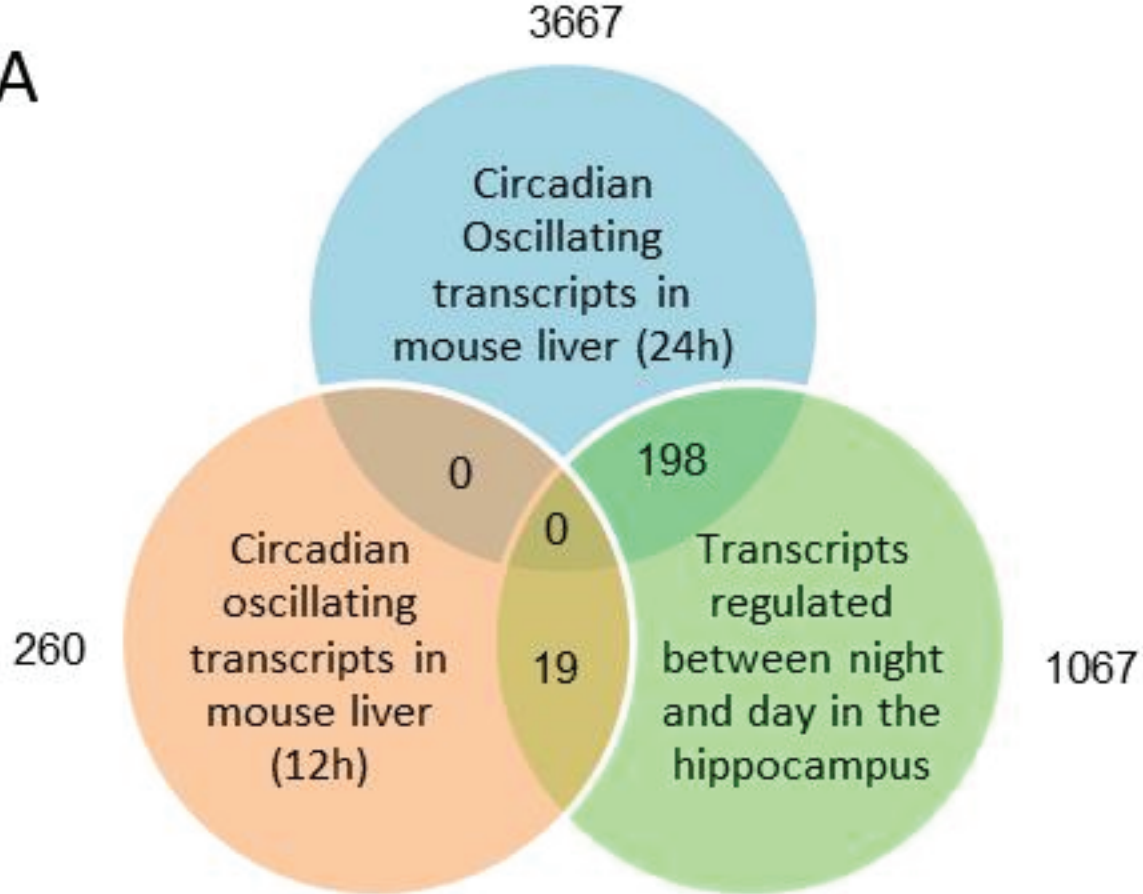

S3B

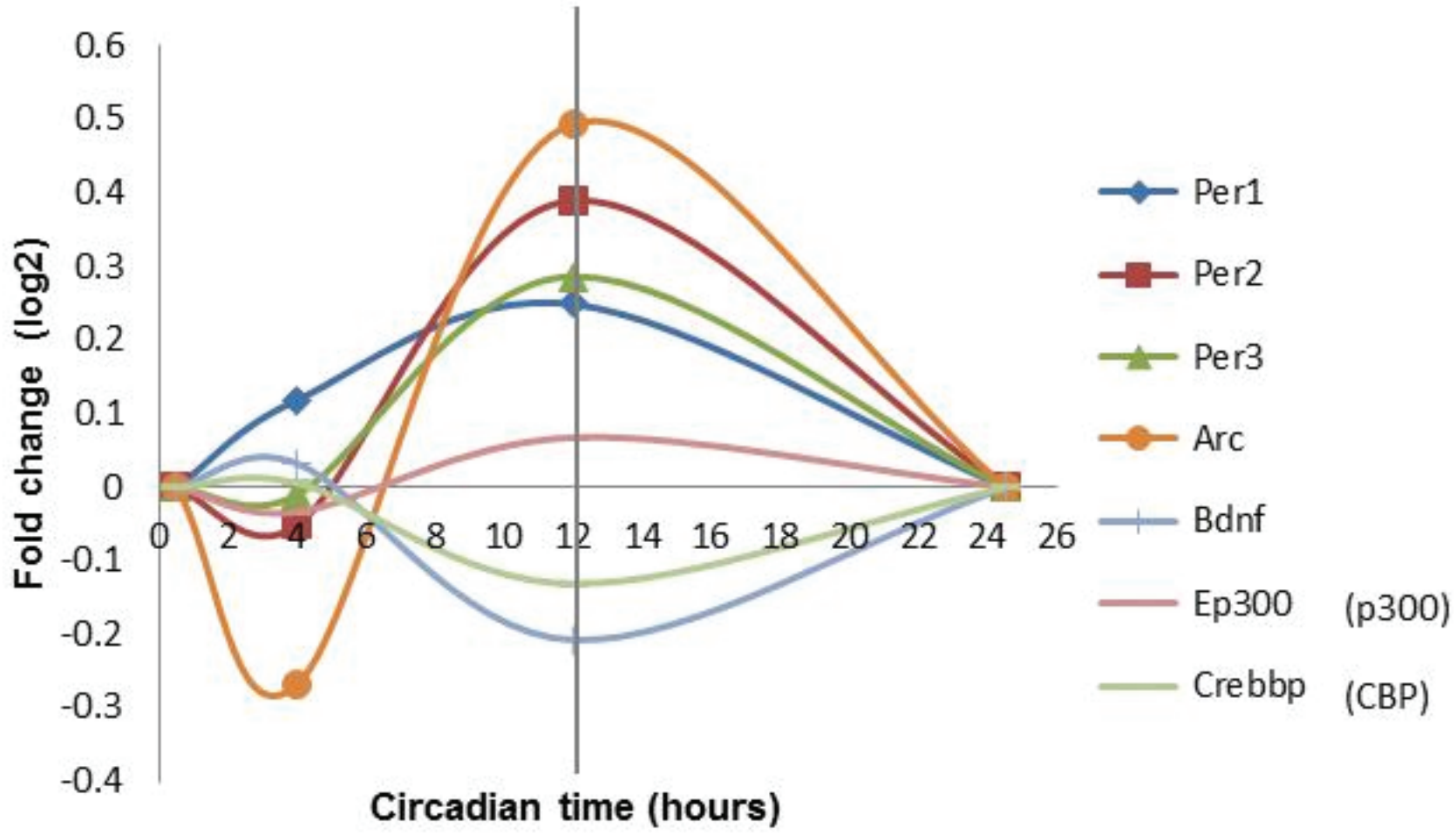

Supplement: Additional file 4 — Regulation of circadian gene expression in the hippocampus. A Overlap of genes regulated by time of day in the hippocampus with known circadian oscillators. Transcripts shown to be regulated in the mouse liver at either 24 or 12 hour periods were extracted from Hughes et al [27]. First, probeset ID mapping between Affymetrix mouse Gene Chip 480_2 and Affymetrix Gene 1.1 ST arrays was performed using DAVID [87], allowing all possible mappings. Overlap between the datasets was performed on the basis of the Affymetrix 1.1 Gene ST array IDs. Number of probe sets in each of the datasets is indicated outside of the Venn diagram. B. Differential expression over 24 hs of known circadian oscillators (Per1, Per2, Per3) and known plasticity related genes (Arc, Bdnf, Ep300 and Crebbp). Fold change (log2 scale) relative to CC30 (Y-axis) vs. time relative to CC30 (X-axis). Twelve hours' time point (CC12) is highlighted and it overlaps with a peak in activity for mice (10 pm). [file 1471-2164-16-S5-S5-S4.pdf]

**A**

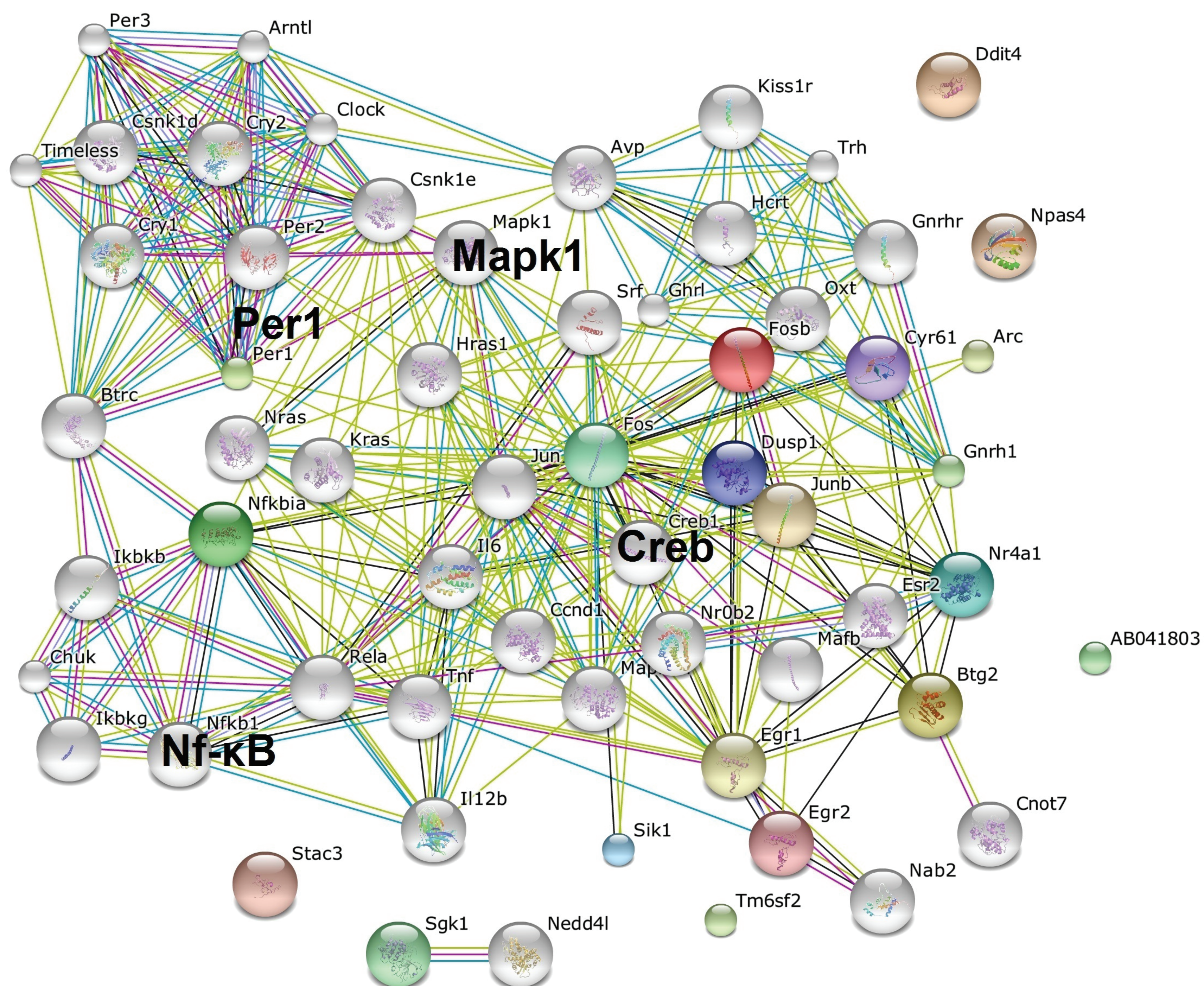

# B

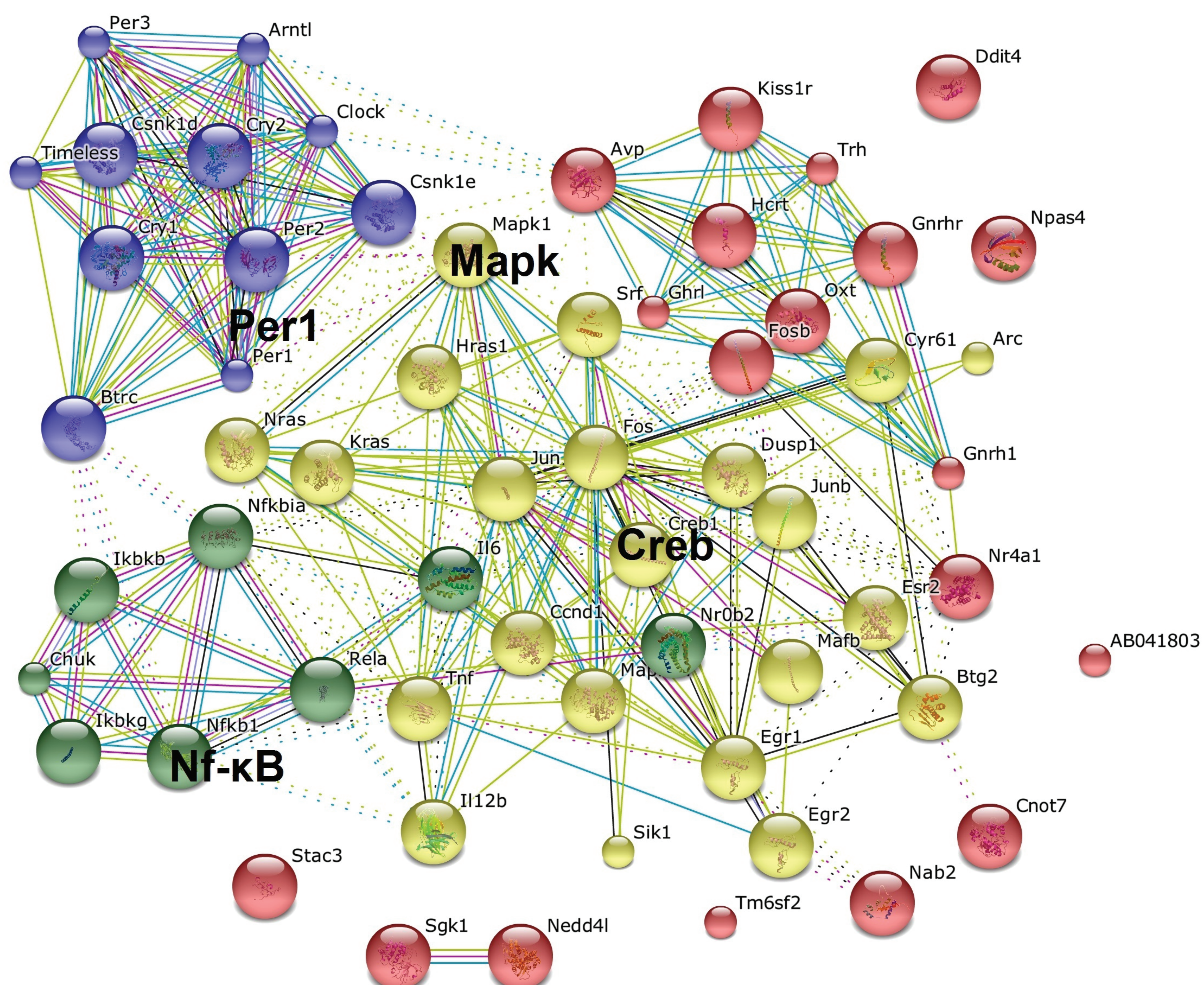

Supplement: Additional file 8 — Regulatory networks induced by memory acquisition and retrieval. Functional interaction analysis of protein coding genes induced at FC30' and RT30' (STRING [80]). A. Interaction network of genes induced at FC30' and RT30'. Only 20 out of the 27 non-intronic probesets up-regulated at FC30' and RT30' (fdr <0.1) mapped to mouse proteins present in the STRING database (colored nodes), 40 additional nodes were incorporated based on predicted interactions (white nodes). Interactions between nodes are represented as colored lines, only medium to high confidence interactions are shown (interaction score > 0.4). Colors represent sources of information as follows: co-expression (black), experiments (magenta), databases (blue) and PubMed text-mining (green). Interaction score > 0.4 for all shown interactions. B Results of K-mean clustering (k = 4) of the interaction network shown above. Clusters are color coded, red nodes representing unclustered nodes. Three clear clusters are present in the network: a MAPK/CREB cluster (yellow), an Nf-κB cluster (in green) and a Per1 cluster (in blue). Interactions within clusters are represented by solid colored lines, while interactions among clusters are represented by dashed colored lines. Colors represent sources of information as previously described. [file 1471-2164-16-S5-S5-S8.pdf]

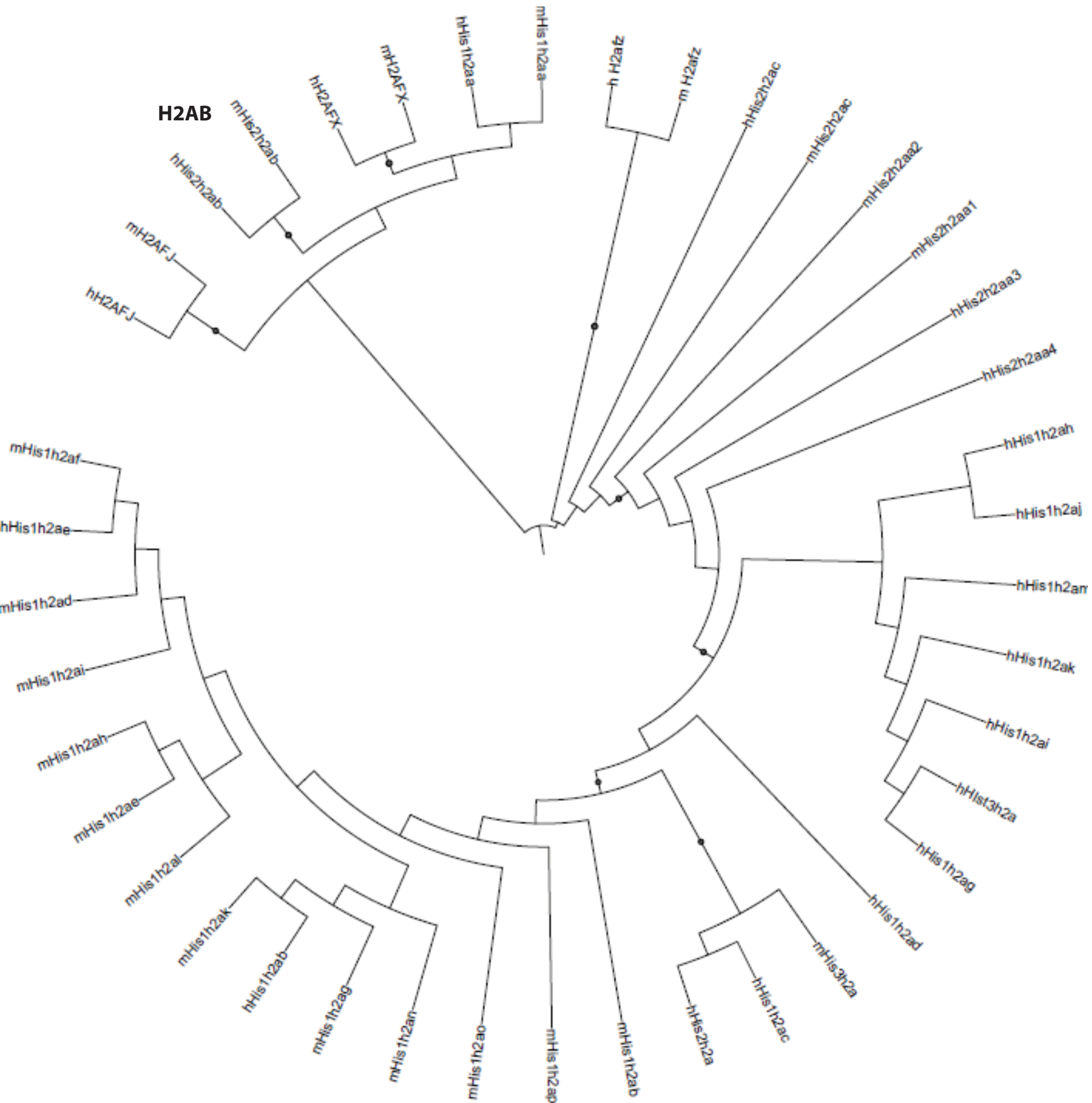

Supplement: Additional file 10 — Molecular Phylogeny of human and mouse orthologs of H2AB (Hist2h2ab). Mouse Hist2h2ab was mapped to orholog group OG5_126570 using OrthoMCL [83]. Human and mouse Ensembl sequences from the group were aligned using T-coffee [88] and phylogeny reconstruction was performed using PhyML [85] using aLRT likelihood to calculate branch support. Gene names are displayed, m depicts mouse sequences, h depicts human sequences. Black dots indicate braches with >0.8 support. [file 1471-2164-16-S5-S5-S10.pdf]
