# Supplementary material for: Memory acquisition and retrieval impact different epigenetic processes that regulate gene expression
Source: BMC Genomics. 2015 May 26;16(Suppl 5):S5. doi: 10.1186/1471-2164-16-S5-S5 (PMC4460846; doi:10.1186/1471-2164-16-S5-S5)
Supplement: Additional file 2 — Functional clustering of genes whose scores are correlated between PC1 and PC2. DAVID functional clustering [87] for the 172 probe-sets (58 genes) whose scores are correlated between PC1 and PC2 (Figure S1C). Enrichment scores (EASE) for functional clusters are calculated as the negative logarithm of the geometric mean of the p-values for individual terms in the cluster including only functional terms with a p-value <0.05 and at least 3 genes and using a cutoff of EASE >1.3 to define enriched clusters (p-value geometric mean <0.05). Only one cluster was identified above the cutoff, with EASE = 3.41, containing 4 functional terms all related to pheromone function. Number of genes that belong to each functional term as well as the term enrichment p-value is displayed on the right. Horizontal bars represent the proportion of the total genes in the list that belong to the individual functional term. [file 1471-2164-16-S5-S5-S2.pdf]

Annotation Cluster 1	Enrichment Score: 3.41			Count	P_Value
GOTERM_MF_FAT	pheromone receptor activity	RT		5	9.4E-6
SP_PIR_KEYWORDS	receptor	RT		8	7.6E-4
GOTERM_BP_FAT	G-protein coupled receptor protein signaling pathway	RT		8	7.6E-4
GOTERM_BP_FAT	cell surface receptor linked signal transduction	RT		8	4.2E-3

Additional file 2, *Peixoto et al.*
