# Supplementary material for: Memory acquisition and retrieval impact different epigenetic processes that regulate gene expression
Source: BMC Genomics. 2015 May 26;16(Suppl 5):S5. doi: 10.1186/1471-2164-16-S5-S5 (PMC4460846; doi:10.1186/1471-2164-16-S5-S5)
Supplement: Additional file 6 — Functional clustering of genes upregulated at FC30' and RT30'. DAVID functional clustering [87] for the 27 genes that are up-regulated at FC30' and RT30' at an fdr<0.1. Enrichment scores (EASE) for functional clusters are calculated as the negative logarithm of the geometric mean of the enrichment p-values for individual functional terms in the cluster. Only terms with p-value <0.05 with and at least 3 genes are included in the clustering. Only clusters with EASE >1.3 are considered enriched clusters (p-value geometric mean <0.05). Only one cluster with enrichment score of 2.63, containing 30 functional terms was identified. All functional terms are related to regulation of transcription. Number of genes in each cluster as well as individual enrichment p-values for each functional term is displayed. [file 1471-2164-16-S5-S5-S6.pdf]

Annotation Cluster 1	Enrichment Score: 2.63			Count	P_Value
GOTERM_BP_FAT	response to organic substance	RT		7	3.4E-5
GOTERM_BP_FAT	regulation of transcription	RT		11	1.8E-4
SP_PIR_KEYWORDS	dna-binding	RT		8	4.1E-4
GOTERM_MF_FAT	protein dimerization activity	RT		5	5.1E-4
SP_PIR_KEYWORDS	nucleus	RT		12	5.7E-4
SP_PIR_KEYWORDS	transcription regulation	RT		8	7.6E-4
GOTERM_BP_FAT	regulation of transcription from RNA polymerase II promoter	RT		6	1.1E-3
GOTERM_BP_FAT	positive regulation of transcription from RNA polymerase II promoter	RT		5	1.2E-3
GOTERM_MF_FAT	transcription factor activity	RT		6	1.3E-3
GOTERM_MF_FAT	transcription regulator activity	RT		7	1.5E-3
SP_PIR_KEYWORDS	Transcription	RT		8	1.6E-3
GOTERM_BP_FAT	regulation of transcription, DNA-dependent	RT		8	1.8E-3
GOTERM_BP_FAT	regulation of RNA metabolic process	RT		8	2.0E-3
GOTERM_MF_FAT	DNA binding	RT		8	2.0E-3
GOTERM_BP_FAT	positive regulation of transcription, DNA-dependent	RT		5	2.1E-3
GOTERM_BP_FAT	positive regulation of RNA metabolic process	RT		5	2.1E-3
SP_PIR_KEYWORDS	DNA binding	RT		4	2.4E-3
GOTERM_BP_FAT	positive regulation of transcription	RT		5	3.3E-3
GOTERM_BP_FAT	positive regulation of gene expression	RT		5	3.7E-3
GOTERM_BP_FAT	positive regulation of nucleobase, nucleoside, nucleotide and nucleic acid metabolic process	RT		5	4.3E-3
GOTERM_BP_FAT	positive regulation of nitrogen compound metabolic process	RT		5	4.8E-3
GOTERM_BP_FAT	positive regulation of macromolecule biosynthetic process	RT		5	4.9E-3
GOTERM_BP_FAT	positive regulation of cellular biosynthetic process	RT		5	5.6E-3
GOTERM_BP_FAT	positive regulation of biosynthetic process	RT		5	5.8E-3
GOTERM_BP_FAT	transcription	RT		8	5.9E-3
GOTERM_MF_FAT	protein heterodimerization activity	RT		3	8.4E-3
GOTERM_BP_FAT	positive regulation of macromolecule metabolic process	RT		5	9.0E-3
GOTERM_MF_FAT	sequence-specific DNA binding	RT		4	2.3E-2
GOTERM_MF_FAT	transcription activator activity	RT		3	3.5E-2
SP_PIR_KEYWORDS	zinc-finger	RT		5	3.7E-2

Additional file 6, *Peixoto et al.*
