# Supplementary material for: Memory acquisition and retrieval impact different epigenetic processes that regulate gene expression
Source: BMC Genomics. 2015 May 26;16(Suppl 5):S5. doi: 10.1186/1471-2164-16-S5-S5 (PMC4460846; doi:10.1186/1471-2164-16-S5-S5)
Supplement: Additional file 7 — Functional clustering of genes down-regulated at FC30' and RT30'. DAVID functional clustering [87] for genes that are down-regulated at FC30' or RT30' (fdr<0.1). Enrichment scores (EASE) for functional clusters are calculated as the negative logarithm of the geometric mean of the enrichment p-values for individual functional terms in the cluster. Only terms with p-value <0.05 with and at least 3 genes are included in the clustering. Only clusters with EASE >1.3 are considered enriched clusters (p-value geometric mean <0.05). A. Functional clustering for the set of 63 genes (72 probe-sets) downregulated at FC30' but not at RT30'. Only one cluster with enrichment score of 2.73, containing 20 functional terms was identified. All functional terms are related to chromatin assembly. B Functional clustering for the set of 84 genes (107 probe-sets) downregulated at RT30' but not at FC30'. Only one cluster with enrichment score of 1.58, containing 6 functional terms was identified. All functional terms are related to RNA processing. Number of genes that belong to each functional term as well as the term enrichment p-value is displayed on the right. Horizontal bars represent the proportion of the total genes in the list that belong to the individual functional term. [file 1471-2164-16-S5-S5-S7.pdf]

A

Annotation Cluster 1	Enrichment Score: 2.78			Count	P_Value	Benjamini
SP_PIR_KEYWORDS	citrullination	RT		4	3.8E-6	3.8E-4
SP_PIR_KEYWORDS	nucleosome core	RT		4	8.6E-5	4.3E-3
GOTERM_BP_FAT	nucleosome assembly	RT		4	3.5E-4	8.4E-2
GOTERM_BP_FAT	chromatin assembly	RT		4	3.8E-4	4.6E-2
GOTERM_BP_FAT	nucleosome organization	RT		4	4.0E-4	3.2E-2
GOTERM_BP_FAT	protein-DNA complex assembly	RT		4	4.0E-4	3.2E-2
KEGG_PATHWAY	Systemic lupus erythematosus	RT		4	8.3E-4	7.5E-3
SP_PIR_KEYWORDS	methylation	RT		5	8.9E-4	2.9E-2
GOTERM_BP_FAT	DNA packaging	RT		4	9.2E-4	5.5E-2
GOTERM_BP_FAT	chromatin assembly or disassembly	RT		4	1.1E-3	5.5E-2
SP_PIR_KEYWORDS	chromosomal protein	RT		4	2.4E-3	5.8E-2
GOTERM_BP_FAT	chromatin organization	RT		5	2.8E-3	1.1E-1
SP_PIR_KEYWORDS	acetylation	RT		12	3.7E-3	7.2E-2
GOTERM_BP_FAT	chromosome organization	RT		5	6.9E-3	2.2E-1
GOTERM_BP_FAT	cellular macromolecular complex assembly	RT		4	8.0E-3	2.2E-1
GOTERM_BP_FAT	cellular macromolecular complex subunit organization	RT		4	1.1E-2	2.6E-1
SP_PIR_KEYWORDS	isopeptide bond	RT		4	1.7E-2	2.5E-1
SP_PIR_KEYWORDS	nucleus	RT		14	2.4E-2	2.9E-1
GOTERM_BP_FAT	macromolecular complex assembly	RT		4	2.6E-2	4.8E-1
GOTERM_BP_FAT	macromolecular complex subunit organization	RT		4	3.2E-2	5.2E-1

B

Annotation Cluster 1	Enrichment Score: 1.58			Count	P_Value
GOTERM_BP_FAT	RNA splicing	RT		4	1.6E-2
SP_PIR_KEYWORDS	mrna splicing	RT		4	1.8E-2
GOTERM_BP_FAT	RNA processing	RT		5	2.7E-2
GOTERM_BP_FAT	mRNA processing	RT		4	3.1E-2
SP_PIR_KEYWORDS	mrna processing	RT		4	3.2E-2
GOTERM_BP_FAT	mRNA metabolic process	RT		4	4.5E-2

Additional file 7, *Peixoto et al.*
