# Supplementary material for: Memory acquisition and retrieval impact different epigenetic processes that regulate gene expression
Source: BMC Genomics. 2015 May 26;16(Suppl 5):S5. doi: 10.1186/1471-2164-16-S5-S5 (PMC4460846; doi:10.1186/1471-2164-16-S5-S5)
Supplement: Additional file 9 — Multiple sequence alignment of the c-terminal portion of Histone 2A. Mouse Hist2h2ab (H2AB) was mapped to orholog group OG5_126570 using OrthoMCL [83]. Human and mouse Ensembl sequences from the group were aligned using T-coffee [88]. Positions 69-145 are displayed, since no differences between H2AB and other H2A sequences are observed in the N-terminus. Gene names are displayed, m depicts mouse sequences, h depicts human sequences. Residues conserved in all sequences are not-color coded. H2AB and the residues unique to this variant are highlighted in green. Magenta: AA residues for which only one histone variant differs from others. Orange-yellow shades: AA residues for which several H2A sequences differ from each other. The red box highlights the peptide used to identify H2AB in the quantitative proteomics analysis. [file 1471-2164-16-S5-S5-S9.pdf]

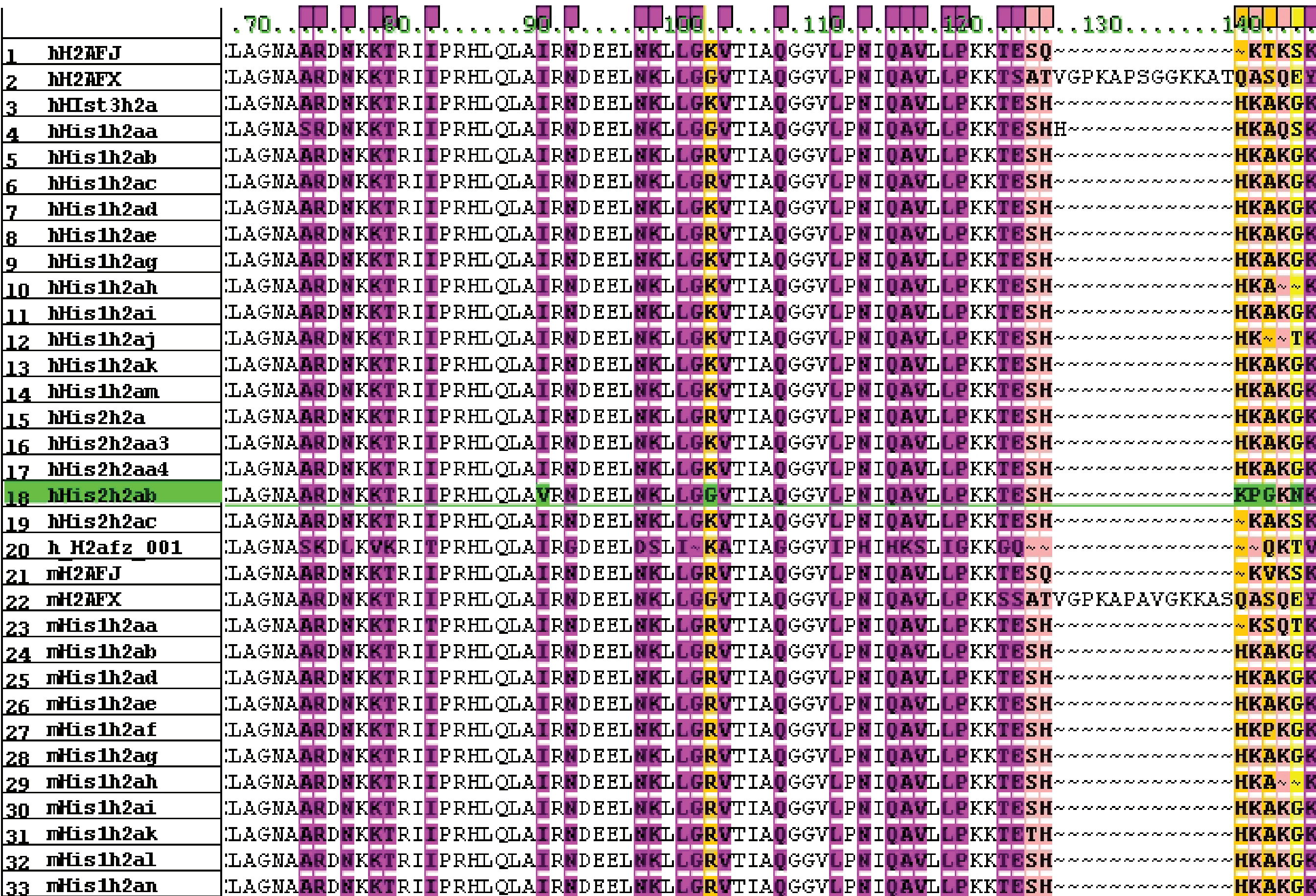

Additional file 9, *Peixoto et al.*
