# Supplementary material for: Memory acquisition and retrieval impact different epigenetic processes that regulate gene expression
Source: BMC Genomics. 2015 May 26;16(Suppl 5):S5. doi: 10.1186/1471-2164-16-S5-S5 (PMC4460846; doi:10.1186/1471-2164-16-S5-S5)
Supplement: Additional file 11 — MicroRNAs regulated after acquisition or retrieval of memory in our microarray study. Non-coding RNAs (precursors) regulated at FC30' or RT30' in the microarray study. Values represent fold change relative to control (CC30'). Green background: up-regulated, Red background: down-regulated. Statistical significance is highlighted by font color: fdr <0.01 (in red) or fdr <0.1 (in orange). N.S: not significant differences in gene expression. [file 1471-2164-16-S5-S5-S11.pdf]

Gene	FC30	RT30
Snord14e	1.8	2.2
Snord14d	1.5	1.8
miR-212/132 p	1.4	1.4
miR-505p	1.2	NS
Mirg	NS	1.2
miR-547p	1.1	NS
miR-488p	0.9	NS
miR- 128-2p	0.8	NS
miR-9-1p	0.8	NS
miR-9-2p	NS	0.9
miR-138-2p	NS	0.9
miR-181p	NS	0.9
miR-497p	NS	0.8
miR-219p	NS	0.7

Additional file 11, *Peixoto et al.*
