# Supplementary material for: Memory acquisition and retrieval impact different epigenetic processes that regulate gene expression
Source: BMC Genomics. 2015 May 26;16(Suppl 5):S5. doi: 10.1186/1471-2164-16-S5-S5 (PMC4460846; doi:10.1186/1471-2164-16-S5-S5)
Supplement: Additional file 12 — Assays IDs or primer sequences for genes tested by qPCR. [file 1471-2164-16-S5-S5-S12.pdf]

Gapdh	**Mm99999915_g1 (Taqman)**
Fos	**Mm00487425_m1 (Taqman)**
Sox18	**Mm00656049_gH (Taqman)**
Btg2	**Mm00476162_m1 (Taqman)**
Sik1	**Mm00440317_m1 (Taqman)**
Rbfox1 (A2bp1)	**Mm00480615_m1 (Taqman)**
Hist2h2ab	**Mm01613463_s1 (Taqman)**
Per1 (1B)	**Mm01325257_m1 (Taqman)**
Per 1 (18)	**Mm00501813_m1 (Taqman)**
Snord68	**Hs_SNORD68_11 (miScript control)**
miR-132	**Mm_miR-132*_1(miSCRIPT)**
miR-212	**Mm_miR-212-3p_1(miSCRIPT)**
miR-410	**Mm_miR-410*_1(miSCRIPT)**
miR-219	**Mm_miR-219-2-3p_1(miSCRIPT)**
Snord14d	**CCACCAGAAATGGTGTGTTG**
Snord14e	**CACCAGAACGCAAGGCAGTGTTG**

Additional file 12, *Peixoto et al.*
